# A multicountry study to establish rates for pregnancy and neonatal outcomes in low- and middle-income regions

**DOI:** 10.1186/s12884-025-08012-1

**Published:** 2026-01-14

**Authors:** Antonella Nadia Tullio, Emad Yanni, Conrado Milani Coutinho, Lavitha Sivapatham, Christine Mui Fong Lee, Sridevi Pallem, Yongjia Pu, Agnes Akawung, Joon Hyung Kim, Ouzama Henry, Marisa Marcia Mussi-Pinhata, Khatija Ahmed, Clara del Carmen Flores Acosta, Osvaldo Reyes, Ivonne Abadía de Regalado, Diana Andrea Arias Fernandez, Linda Aurpibul, Sri Wahyu Taher, Juliana Caccavo, Ana Ceballos, Chenchit Pichailuck, Ulises D’Andrea Nores, Tirza De Leon, Mara De Bernardi, Pablo Dieser, Emily Christine D’Silva, Andrea Falaschi, Samantha Fry, Angela Gentile, Ik Hui Teo, Sheena Kotze, Eduardo Lopez-Medina, Ruben Luca, Maria Florencia Lucion, Jacinto Blass III V. Mantaring, Bladimir Marin, Malahleha Moelo, Jorge Pinto, Thanyawee Puthanakit, Maria Fernanda Roa, Maria Teresa Rodriguez Brieschke, Camilo Enrique Rodriguez, Juan Nicolas Rodriguez Niño, Alexandre Vargas Schwarzbold, Alexandra Sierra Garcia, Ruey Soon, Juan Carlos Tinoco, Jesús Arnulfo Velásquez Penagos, Khalequ Zaman, Gaël Dos Santos

**Affiliations:** 1 GSK, Rockville, MD USA; 2 Department of Gynecology and Obstetrics, Hospital das Clínicas da Faculdade de Medicina de Ribeirão Preto, Universidade de São Paulo, Ribeirão Preto, Brazil; 3 Department Obstetrics and Gynecology, Ampang Hospital, Ampang, Selangor Malaysia; 4 Department of Obstetrics and Gynaecology, Sarawak General Hospital, Kuching, Malaysia; 5 Keyrus Life Science C/O GSK, Wavre, Belgium; 6 Department of Pediatrics, Ribeirão Preto Medical School, University of São Paulo, Ribeirão Preto, Brazil; 7 Setshaba Research Centre NPC, Soshanguve, South Africa; 8 Department of Paediatrics & Child Health, School of Clinical Medicine, University of the Witwatersrand, Johannesburg, South Africa; 9 Department of Medical Microbiology, Faculty of Health Sciences, University of Pretoria, Pretoria, South Africa; 10 Dr. José E. González University Hospital, Autonomous University of Nuevo León, Monterrey, Nuevo León, Mexico; 11 Santo Tomás Hospital, Panama City, Panama; 12 Centro de Vacunación Internacional S.A. (CEVAXIN), La Chorrera, Panama; 13 Sistema Nacional de Investigadores (SNI), Panama City, Panama; 14 Policentro de Salud de Juán Diaz, Juán Diaz, Panama City, Panama; 15 Hospital San José, Bogotá, Colombia; 16 Research Institute for Health Sciences, Chiang Mai University, Chiang Mai, Thailand; 17 Simpang Kuala Health Clinic, Alor Setar, Malaysia; 18 Donación Francisco Santojanni Hospital, Buenos Aires, Argentina; 19 Instituto Medico Rio Cuarto, Rio Cuarto, Còrdoba, Argentina; 20 Department of Obstetrics and Gynaecology, Faculty of Medicine Siriraj Hospital, Mahidol University, Bangkok, Thailand; 21 Maternity Hospital José Domingo De Obaldia, San Pablo Viejo, Panama; 22 Dr. Ramon Carrillo Hospital, Mendoza, Argentina; 23 Dr. Diego Paroissien Hospital, Mendoza, Argentina; 24 Department of Paediatrics and Child Health, Family Centre for Research with Ubuntu, Stellenbosch University, Cape Town, South Africa; 25 Department of Epidemiology, Ricardo Gutiérrez Children’s Hospital, University of Bueno Aires, Buenos Aires, Argentina; 26 Hospital Ampang, Kuala Lumpur, Malaysia; 27 Synexus Stanza Clinical Research Centre, Gauteng, Pretoria South Africa; 28 Centro de Estudios de Infectología Pediátrica (CEIP), Cali, Colombia; 29 Department of Pediatrics, Universidad del Valle, Cali, Colombia; 30 Clínica Imbanaco, Grupo Quironsalud, Cali, Colombia; 31 Hospital F.F. Santojanni, Buenos Aires, Argentina; 32 Epidemiology Department, Hospital de Niños Dr. Ricardo Gutiérrez, Buenos Aires, Argentina; 33 University of the Philippines, Philippine General Hospital, Manila, Philippines; 34 Hospital San José, Bogotá, Colombia; 35 Federal University of Minas Gerais, Belo Horizonte, Brazil; 36 Department of Pediatrics, Center of Excellence for Pediatric Infectious Diseases and Vaccines, Faculty of Medicine, Chulalongkorn University, Bangkok, Thailand; 37 Department of Pediatrics, University Hospital Fundación Santa Fe de Bogotá, Bogotá, Colombia; 38 Department of Gynecology and Obstetrics, University Hospital Fundación Santa Fe de Bogotá, Bogotá, Colombia; 39 School of Medicine, University of the Andes, Bogotá, Colombia; 40 Centro de Pesquisa Clínica, Hospital Universitário de Santa Maria (HUSM-EBSERH), Universidade Federal de Santa Maria, Santa Maria, Rio Grande Do Sul Brazil; 41 Centro de Estudios en Infectología Pediátrica (CEIP), Cali, Colombia; 42 Department of Pediatrics, Universidad del Valle, Valle del Cauca, Cali, Colombia; 43 Clínica Imbanaco, Grupo Quironsalud, Cali, Colombia; 44 Department of Obstetrics and Gynecology, Sabah Women’s and Children’s Hospital, Kota Kinabalu, Malaysia; 45 General Hospital of Durango, Durango, Mexico; 46 San Vicente Fundación University Hospital, Medellin, Colombia; 47 International Centre for Diarrhoeal Disease Research (icddr,b), Dhaka, Bangladesh; 48 GSK, Wavre, Belgium; 49 AstraZeneca, Gaithersburg, MD USA; 50 AbbVie, MD, USA; 51 Independent researcher, Wavre, Belgium; 52 Synergy Biomed Research Institute, East London, South Africa; 53 Dynavax Technologies, Emeryville, CA United States

**Keywords:** Pregnancy outcome, Pregnant women, Infant, Newborn, Vaccination, Evidence gaps, LMIC

## Abstract

**Background:**

Maternal vaccines can reduce the burden of diseases for both mother and neonate. The development and implementation of such vaccines require a thorough interpretation of safety data and standardized disease case definitions.

This study aimed to evaluate the current rates for adverse pregnancy outcomes, maternal and neonatal events of interest (EOIs) that will be informative for preparing future phase III clinical trials on maternal vaccines conducted in low- and middle-income countries and further assisting in safety data interpretation (e.g., monitoring potential safety signals in clinical trials, and/or helping with the causality assessments of adverse events following immunization).

**Methods:**

We performed a prospective cohort study on healthy 18–45-year-old women, with singleton, low-risk pregnancies, with a gestational age of ≥24^0/7^ weeks at enrollment and ≤27^6/7^ weeks at first visit, and their neonates. The study was conducted between 2019–2021 in 10 countries that were considered low- and middle-income countries by the World Bank Group at the time the study was designed. All pregnancy-related outcomes and maternal EOIs occurring from enrollment up to 42 days post-delivery and neonatal EOIs occurring within the first 28 days after birth were recorded.

**Results:**

Of 2311 pregnant women and 2181 neonates enrolled, 2222 and 2094 were included in the analyses, respectively. Most livebirths (2088 [94.0%]) were without apparent congenital anomalies. Preterm delivery (166 [7.5%]), non-reassuring fetal status (137 [6.2%]), and hypertensive disorders of pregnancy (125 [5.6%]) were the most frequently reported EOIs related to pregnancy. The most frequent neonatal EOIs were low birthweight (including very low birthweight) (156 [7.4%]), preterm birth (141 [6.7%]), small for gestational age (111 [5.3%]), and congenital anomalies (103 [4.9%], including mainly major external structural defects, as well as detected internal and functional defects). Overall, there were similar frequencies in pregnancy outcomes, pregnancy-related and neonatal EOIs across countries, although some variation in the reporting rate was noted.

**Conclusion:**

This multicountry study contributes to establishing the most recent background rates for pregnancy outcomes, maternal and neonatal EOIs in low- and middle-income regions. The clinical relevance in the context of the safety assessment in future trials will be applied.

**Trial registration:**

NCT03614676 (03/08/2018).

**Supplementary Information:**

The online version contains supplementary material available at 10.1186/s12884-025-08012-1.

## Background

Pregnant women and neonates are known to be susceptible to several vaccine-preventable infectious diseases and their complications [1, 2]. Maternal immunization is expected to reduce the burden of infectious diseases [3], including pediatric in-hospital deaths [4]. The World Health Organization and several regulatory bodies currently recommend routine maternal vaccination against influenza, pertussis, diphtheria, tetanus, and COVID-19 [5–9]. These vaccines were shown to be effective, while maintaining an acceptable safety profile [3, 10–13]. In addition, influenza vaccination in pregnant women is known to decrease the number of hospitalizations for acute respiratory infections in their ≤ 6-months-old children [14]. In several countries, other vaccines (e.g., against hepatitis A/B, poliovirus, or yellow fever) are also recommended to pregnant women who are at risk [9, 15]. Further development of maternal and pediatric vaccines can have health, economic, and social benefits [14].

Assessing the safety of maternal vaccines requires careful monitoring of both maternal, perinatal, and neonatal outcomes. Extensive knowledge on background rates of clinically important events of interest (EOIs) is paramount to monitor and assess vaccine safety data, investigate potential safety signals, and/or contribute to the causality assessment of adverse events following immunization throughout vaccine development and post-licensure [13, 16].

Worldwide, nearly 90% of pregnancies and > 60% of preterm births occur in low- and middle-income countries (LMICs) [17, 18], where proper access to prenatal care is sometimes lacking [19] and frequent complications during pregnancy lead to high risk of maternal and neonatal EOIs [20]. Data on maternal and neonatal outcomes are seldom routinely collected in LMICs, often with inconsistencies in diagnosis and reporting, making the conduct of clinical trials on maternal vaccination challenging [21].

In view of supporting a pivotal clinical trial for the respiratory syncytial virus (RSV) maternal program [22], we performed a multicountry, prospective study in LMICs, aiming to provide more robust data on the incidence of maternal and neonatal EOIs that might be considered adverse events should they occur after vaccination in healthy pregnant women or their neonates (Supplementary Fig. 1).

## Methods

This prospective, cohort study (without administration of medicinal products) took place from May 2019 until October 2021 in 37 centers across 10 countries (two in Bangladesh; three in the Philippines, three in Thailand, three in South Africa, and three in Brazil; four in Malaysia, four in Mexico, and four in Panama; five in Argentina; six in Colombia). At the time of the study design all 10 countries were considered LMICs by the World Bank Group [23].

### Cohort composition

The study was conducted in two cohorts: maternal participants and their neonates. Healthy, pregnant women, who were 18–45 years of age, with singleton pregnancies, and with a pre-pregnancy body mass index (BMI) between 18.5 and 39.9 kg/m^2^ were enrolled in the study. These women had a gestational age of ≥ 24^0/7^ weeks at screening and ≤ 27^6/7^ weeks at first visit, as established mainly by ultrasound examination and, if not possible, by last menstruation date assessed according to the Global Alignment of Immunization safety Assessment (GAIA) consortium case definition [24], and a pregnancy considered low-risk. Low-risk pregnancies correspond to pregnancies without complications or other factors that might increase the risk for complications ([25], Supplementary Table 1), based on medical and obstetric history and clinical findings during the pregnancy.

After the delivery, all liveborn neonates were eligible for enrollment in the neonatal cohort. Full inclusion and exclusion criteria are given in Supplementary Table 1.

**Fig. 1:**
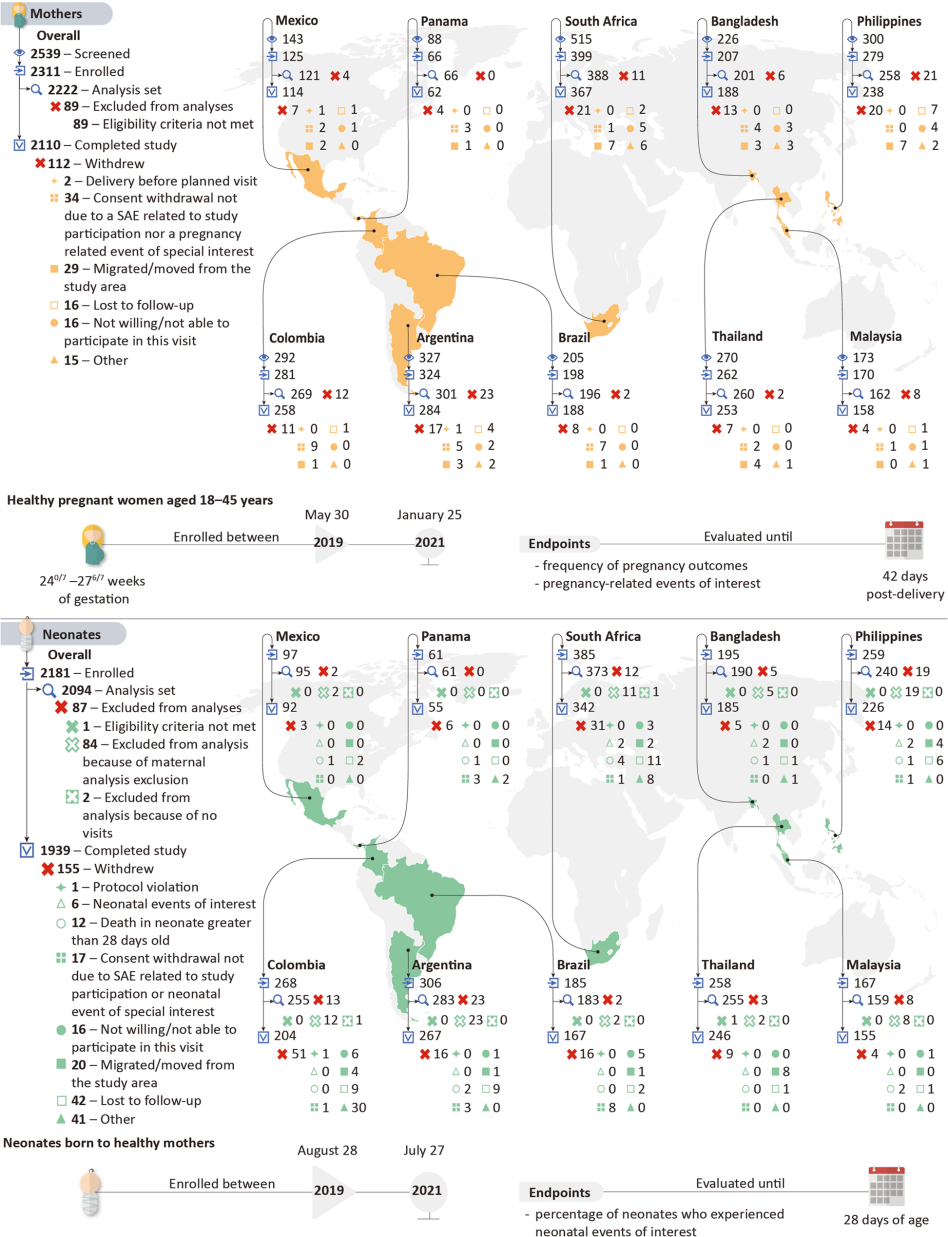
Participant flowchart for mothers and neonates SAE, serious adverse event Note: Neonatal events of interest occur (by definition) between 0 and 28 days after birth, but they might only be detected at a later timepoint; therefore, these events were reported throughout the entire study period

Pregnancy outcomes and pregnancy-related EOIs occurring from enrollment until 42 days post-delivery (including those which were only detectable later) were reported throughout the entire study period (Fig. 1). As some events can still be linked to the pregnancy even if they occur weeks after the delivery date, a 42-day post-partum reporting interval was used, covering the entire postnatal period [26, 27]. During the study follow-up period, all pregnant women completed at least four prenatal visits and one visit at 42 days after the delivery; the mother’s temperature, systolic/diastolic blood pressure, heart rate, respiratory rate at rest, and blood oxygen saturation were measured at every visit. At every prenatal visit, general and obstetric examinations were performed on each pregnant woman, and included the assessment of weight, fetal heart tones, fetal movement, and fundal height. All pregnant women received at least one scan during pregnancy, where fetal morphology was assessed. Maternal blood samples to evaluate hematology/biochemistry parameters were collected at baseline (day 1); the hemoglobin level was also measured at day 56. Urine samples for urine dipstick tests were collected monthly until delivery for maternal participants.

Neonates were followed until one year of age. We reported neonatal EOIs occurring during the first 28 days from birth, as well as those which could be detected only outside of this timeframe (such as internal structural and functional congenital disorders) throughout the entire study period (Fig. 1).

Examination, sample collection, and documentation were performed by qualified and appropriately trained study personnel.

### Outcomes

Primary objectives were to assess the frequency of pregnancy outcomes and pregnancy-related EOIs from the first visit through 42 days postpartum, as well as neonatal EOIs, up to 28 days after delivery (Supplementary Table 2). Secondary objectives were to determine the frequency of these EOIs according to GAIA case definitions [24, 28–46], harmonizing the active monitoring of safety outcomes in mothers and neonates across different countries [47, 48]. GAIA case definitions [48] and their level of diagnostic certainty [49, 50], or standard definitions (Supplementary Table 3), when GAIA case definitions were unavailable, were used to determine the frequencies of pregnancy-related and neonatal EOIs. Where evidence was insufficient, the event was classified as such. Finally, we aimed to determine the risk factors associated with maternal and neonatal EOIs. Other objectives related to this study will be reported elsewhere.

### Statistical analysis

We planned to enroll approximately 2300 pregnant women, leading to 180–270 evaluable participants per country, when assuming a drop-out rate of 10%. This number of participants per country was considered sufficient to establish the rates of common and very common EOIs. The percentages of participants with maternal, fetal, and neonatal EOI for the primary objectives were expected to range from 0 to 35%. Exact 95% confidence intervals (CIs) were calculated for different sample sizes to quantify the precision of estimates.

We conducted the primary analyses on the maternal and neonatal analysis sets, comprising all enrolled participants who met all eligibility criteria up to the time of their censoring (i.e., at study completion or prematurely as drop-out). Neonates in the analysis set were born to mothers in the maternal analysis set.

The frequency of pregnancy outcomes and the percentage of participants with at least one outcome or EOI were computed overall and by country, with exact 95% CIs.

Selected variables (e.g., prenatal smoking exposure, pre-pregnancy BMI, maternal age, cesarean section in previous pregnancy, alcohol consumption during the current pregnancy, predominant geographic ancestry, highest education level of the mother, etc.) were assessed as potential risk factors for pregnancy-related and neonatal EOIs using descriptive statistics (see Risk factors for maternal and neonatal EOIs). Variables with p-value ≤ 0.1 in the univariate logistic regression analysis were included in the multivariate logistic regression model for which a p-value of ≤ 0.05 was considered statistically significant. The univariate logistic regression analysis was not performed for rare EOIs (< 10 events reported).

Missing data (e.g., demographic characteristics, laboratory results, outcomes) were not replaced, as planned in the study protocol, to avoid creating potential biases. For selected variables of interest, the number of missing values was provided as a separate category.

All analyses were performed using SAS v9.4 software (SAS Institute, Cary NC), were descriptive, and no adjustment for multiplicity was applied in the analyses.

### Ethics approval

The study was conducted in accordance with the International Council for Harmonisation guidelines for Good Clinical Practice, the Declaration of Helsinki, and applicable local ethics guidelines. Written informed consent was obtained for every participant. Study documents were approved by Institutional Review Boards/Independent Ethics Committees at each site. The trial is registered at www.clinicaltrials.gov (NCT03614676; 03/08/2018).

## Results

### Study population

Of 2539 screened pregnant women, 226 were considered screening failures (most women [198, 87.6%] did not fulfill the inclusion/exclusion criteria) and two had an invalid consent form or fraudulent data; 2311 were enrolled in the study. Eighty-nine enrolled women did not meet the eligibility criteria and were excluded from analyses. The analysis set included 2222 pregnant women; 2110 completed the study. Eighty-five (3.8%) participants in the analysis set did not attend the delivery visit and therefore no outcome was reported for their pregnancies. Of the 2181 singleton neonates born to mothers in the enrolled set, 87 were excluded from analyses (for 84, their mothers were not included in the analysis set, one did not meet eligibility criteria, and for two neonates no study visits were done) and 1939 completed the study, with loss to follow-up being the most frequent withdrawal reason (Fig. 1).

Most maternal participants were aged between 18 and 34 years (86.3%), lived in urban areas (77.1%), were non-smokers (94.1%); 39.8% were Asian, and 14.1% of the participants had a cesarean section in a previous pregnancy (Table 1). Intrauterine fetal growth assessment was appropriate for gestational age in 99.1% of cases. The mean gestational age of the neonates was 38.5 ± 1.7 weeks and the mean 5-minute Apgar score was 9.4 ± 0.8 (Table 2). Overall, demographic and baseline characteristics were similar between mothers and neonates in the enrolled (Supplementary Table 4) and the analysis sets.

**Table 1:** Baseline characteristics for maternal participants (maternal analysis set)

Characteristics	Bangladesh (*N* = 201)	Malaysia (*N* = 162)	Philippines (*N* = 258)	Thailand (*N* = 260)	South Africa (*N* = 388)	Argentina (*N* = 301)	Brazil (*N* = 196)	Colombia (*N* = 269)	Mexico (*N* = 121)	Panama (*N* = 66)	Overall (*N* = 2222)
Age category, n (%)											
18–34 years	194 (96.5)	124 (76.5)	217 (84.1)	196 (75.4)	362 (93.3)	244 (81.1)	173 (88.3)	237 (88.1)	109 (90.1)	61 (92.4)	1917 (86.3)
35–39 years	5 (2.5)	28 (17.3)	31 (12.0)	54 (20.8)	26 (6.7)	49 (16.3)	21 (10.7)	24 (8.9)	9 (7.4)	4 (6.1)	251 (11.3)
≥40 years	2 (1.0)	10 (6.2)	10 (3.9)	10 (3.8)	0 (0.0)	8 (2.7)	2 (1.0)	8 (3.0)	3 (2.5)	1 (1.5)	54 (2.4)
Ethnicity, n (%)											
African/African American	0 (0.0)	0 (0.0)	0 (0.0)	0 (0.0)	271 (69.8)	0 (0.0)	34 (17.3)	58 (21.6)	0 (0.0)	4 (6.1)	367 (16.5)
American Indian/Alaska Native	0 (0.0)	0 (0.0)	0 (0.0)	0 (0.0)	1 (0.3)	0 (0.0)	0 (0.0)	4 (1.5)	41 (33.9)	2 (3.0)	48 (2.2)
Asian	201 (100)	162 (100)	258 (100)	260 (100)	0 (0.0)	0 (0.0)	1 (0.5)	0 (0.0)	0 (0.0)	0 (0.0)	882 (39.8)
White^a^	0 (0.0)	0 (0.0)	0 (0.0)	0 (0.0)	0 (0.0)	301 (100)	83 (42.4)	0 (0.0)	1 (0.8)	0 (0.0)	385 (17.3)
Other	0 (0.0)	0 (0.0)	0 (0.0)	0 (0.0)	116 (29.9)	0 (0.0)	78 (39.8)	207 (77.0)	79 (65.3)	60 (90.9)	540 (24.3)
Highest education level^b^, n (%)											
Less than primary	23 (11.4)	0 (0.0)	1 (0.4)	1 (0.4)	1 (0.3)	2 (0.7)	10 (5.1)	0 (0.0)	0 (0.0)	1 (1.5)	39 (1.8)
Primary	25 (12.4)	5 (3.1)	12 (4.7)	1 (0.4)	5 (1.3)	14 (4.7)	8 (4.1)	21 (7.8)	3 (2.5)	1 (1.5)	95 (4.3)
Secondary	119 (59.2)	89 (54.9)	154 (59.7)	76 (29.1)	301 (77.6)	202 (67.1)	149 (76.0)	176 (65.5)	47 (38.8)	48 (72.7)	1361 (61.2)
Short cycle tertiary	0 (0.0)	38 (23.5)	12 (4.7)	15 (5.8)	59 (15.2)	19 (6.3)	10 (5.1)	19 (7.1)	22 (18.2)	0 (0.0)	194 (8.7)
Bachelor’s	8 (4.0)	24 (14.8)	77 (29.8)	137 (52.7)	15 (3.9)	62 (20.6)	18 (9.2)	37 (13.8)	47 (38.8)	15 (22.7)	440 (19.8)
Master’s	1 (0.5)	5 (3.1)	1 (0.4)	29 (11.2)	0 (0.0)	0 (0.0)	0 (0.0)	11 (4.1)	2 (1.7)	1 (1.5)	50 (2.3)
Not elsewhere classified	25 (12.4)	1 (0.6)	1 (0.4)	1 (0.4)	7 (1.8)	2 (0.7)	1 (0.5)	5 (1.9)	0 (0.0)	0 (0.0)	43 (1.9)
Household environment, n (%)											
Urban	102 (50.7)	143 (88.3)	256 (99.2)	222 (85.4)	60 (15.5)	296 (98.3)	195 (99.5)	262 (97.4)	116 (95.9)	61 (92.4)	1713 (77.1)
Suburban	24 (11.9)	19 (11.7)	1 (0.4)	35 (13.5)	292 (75.3)	3 (1.0)	1 (0.5)	0 (0.0)	4 (3.3)	2 (3.0)	381 (17.1)
Rural	75 (37.3)	0 (0.0)	1 (0.4)	3 (1.2)	36 (9.3)	2 (0.7)	0 (0.0)	7 (2.6)	1 (0.8)	3 (4.5)	128 (5.8)
Smoking status during current pregnancy, n (%)											
Yes, in trimester 1	0 (0.0)	0 (0.0)	10 (3.9)	3 (1.2)	67 (17.3)	17 (5.6)	17 (8.7)	11 (4.1)	3 (2.5)	0 (0.0)	128 (5.8)
Yes, in trimester 2	0 (0.0)	0 (0.0)	5 (1.9)	0 (0.0)	56 (14.4)	3 (1.0)	8 (4.1)	2 (0.7)	1 (0.8)	0 (0.0)	75 (3.4)
No	201 (100)	162 (100)	247 (95.7)	257 (98.8)	319 (82.2)	283 (94.0)	179 (91.3)	258 (95.9)	118 (97.5)	66 (100)	2090 (94.1)
Alcohol consumption during current pregnancy, n (%)											
Yes, in trimester 1	0 (0.0)	0 (0.0)	13 (5.0)	4 (1.5)	51 (13.1)	16 (5.3)	42 (21.4)	13 (4.8)	4 (3.3)	1 (1.5)	144 (6.5)
Yes, in trimester 2	0 (0.0)	0 (0.0)	2 (0.8)	1 (0.4)	25 (6.4)	3 (1.0)	11 (5.6)	0 (0.0)	0 (0.0)	0 (0.0)	42 (1.9)
No	201 (100)	162 (100)	244 (94.6)	255 (98.1)	329 (84.8)	283 (94.0)	149 (76.0)	256 (95.2)	117 (96.7)	65 (98.5)	2061 (92.8)
Living in a country/region with Zika transmission, n (%)											
Yes	200 (99.5)	156 (96.3)	255 (98.8)	256 (98.5)	0 (0.0)	285 (94.7)	195 (99.5)	260 (96.7)	116 (95.9)	65 (98.5)	1788 (80.5)
No	1 (0.5)	6 (3.7)	3 (1.2)	4 (1.5)	388 (100)	16 (5.3)	1 (0.5)	9 (3.3)	5 (4.1)	1 (1.5)	434 (19.5)
Cesarean section (any) in previous pregnancy											
Yes	30 (14.9)	26 (16.0)	18 (7.0)	50 (19.2)	22 (5.7)	52 (17.3)	42 (21.4)	41 (15.2)	29 (24.0)	4 (6.1)	314 (14.1)
No	82 (40.8)	89 (54.9)	131 (50.8)	71 (27.3)	208 (53.6)	107 (35.5)	66 (33.7)	102 (37.9)	29 (24.0)	38 (57.6)	923 (41.5)
N/A	78 (38.8)	42 (25.9)	102 (39.5)	120 (46.2)	141 (36.3)	123 (40.9)	72 (36.7)	101 (37.5)	51 (42.1)	21 (31.8)	851 (38.3)
Missing	11 (5.5)	5 (3.1)	7 (2.7)	19 (7.3)	17 (4.4)	19 (6.3)	16 (8.2)	25 (9.3)	12 (9.9)	3 (4.5)	134 (6.0)

*N* number of participants, *n* (%) number (percentage) of participants in a given category, *N/A* not applicable

^a^White-Arabic/North African ethnicity and White-Caucasian/European ethnicity

^b^Bachelor’s degree or higher

**Table 2:** Characteristics of neonates at birth (neonatal analysis set)

Characteristics	Bangladesh (*N*=190)	Malaysia (*N*=159)	Philippines (*N*=240)	Thailand (*N*=255)	South Africa (*N*=373)	Argentina (*N*=283)	Brazil (*N*=183)	Colombia (*N*=255)	Mexico (*N*=95)	Panama (*N*=61)	Overall (*N*=2094)
Gestational age at birth											
Mean±SD (weeks)	38.2±1.6	38.2±1.7	38.4±1.5	38.4±1.2	38.7±2.0	38.5±1.7	38.9±1.6	38.6±1.7	38.5±1.5	38.9±1.5	38.5±1.7
Missing data, n	0	0	7	0	1	1	0	0	0	1	10
Male sex, n (%)	107 (56.3)	85 (53.5)	138 (57.5)	133 (52.2)	198 (53.1)	125 (44.2)	94 (51.4)	126 (49.4)	50 (52.6)	33 (54.1)	1089 (52)
Length											
Mean±SD (cm)	47.5±2.3	48.7±2.5	48.9±2.7	49.6±2.0	49.8±3.4	48.9±2.5	49.1±2.1	49.8±2.4	49.8±2.7	50±2.6	49.2±2.7
Missing data, n	15	0	11	0	23	1	0	1	0	1	52
Weight											
Mean±SD (kg)	2.8±0.4	3.0±0.5	2.9±0.4	3.1±0.4	3.1±0.5	3.3±0.5	3.3±0.5	3.2±0.4	3.1±0.5	3.3±0.4	3.1±0.5
Missing data, n	12	0	5	0	8	1	0	0	0	1	27
Apgar score at 5 min											
Mean±SD	9.2±0.9	9.4±0.8	9.0±0.2	9.7±0.5	9.6±0.8	9.3±0.8	9.4±0.8	9.4±1.2	9.1±0.5	9.0±0.1	9.4±0.8
Median (Min–Max)	9.0 (4–10)	9.0 (4–10)	9.0 (7–10)	10 (8–10)	10 (4–10)	9.0 (1–10)	9.0 (5–10)	10 (0–10)	9.0 (7–10)	9.0 (8–9)	9.0 (0–10)
Missing data, n	20	45	21	0	38	2	0	35	0	1	162
Breast feeding, n (%)											
Yes	186 (97.9)	155 (97.5)	230 (95.8)	249 (97.6)	361 (96.8)	280 (98.9)	181 (98.9)	255 (100)	93 (97.9)	58 (95.1)	2048 (97.8)
No	1 (0.5)	1 (0.6)	2 (0.8)	1 (0.4)	3 (0.8)	1 (0.4)	1 (0.5)	0 (0.0)	1 (1.1)	1 (1.6)	12 (0.6)
Missing data, n (%)	3 (1.6)	3 (1.9)	8 (3.3)	5 (2.0)	9 (2.4)	2 (0.7)	1 (0.5)	0 (0.0)	1 (1.1)	2 (3.3)	34 (1.6)
Breast feeding duration											
Mean±SD (months)	11.5±2.1	8.5±4.7	8.7±4.5	8.6±4.2	10.0±3.7	11.1±2.9	8.8±4.2	10.5±3.1	10.2±3.6	10.9±3.0	9.9±3.8

*N* number of participants, *SD* standard deviation, *n (%)* number (percentage) of participants in a given category

### Frequency of pregnancy outcomes

**Fig. 2:**
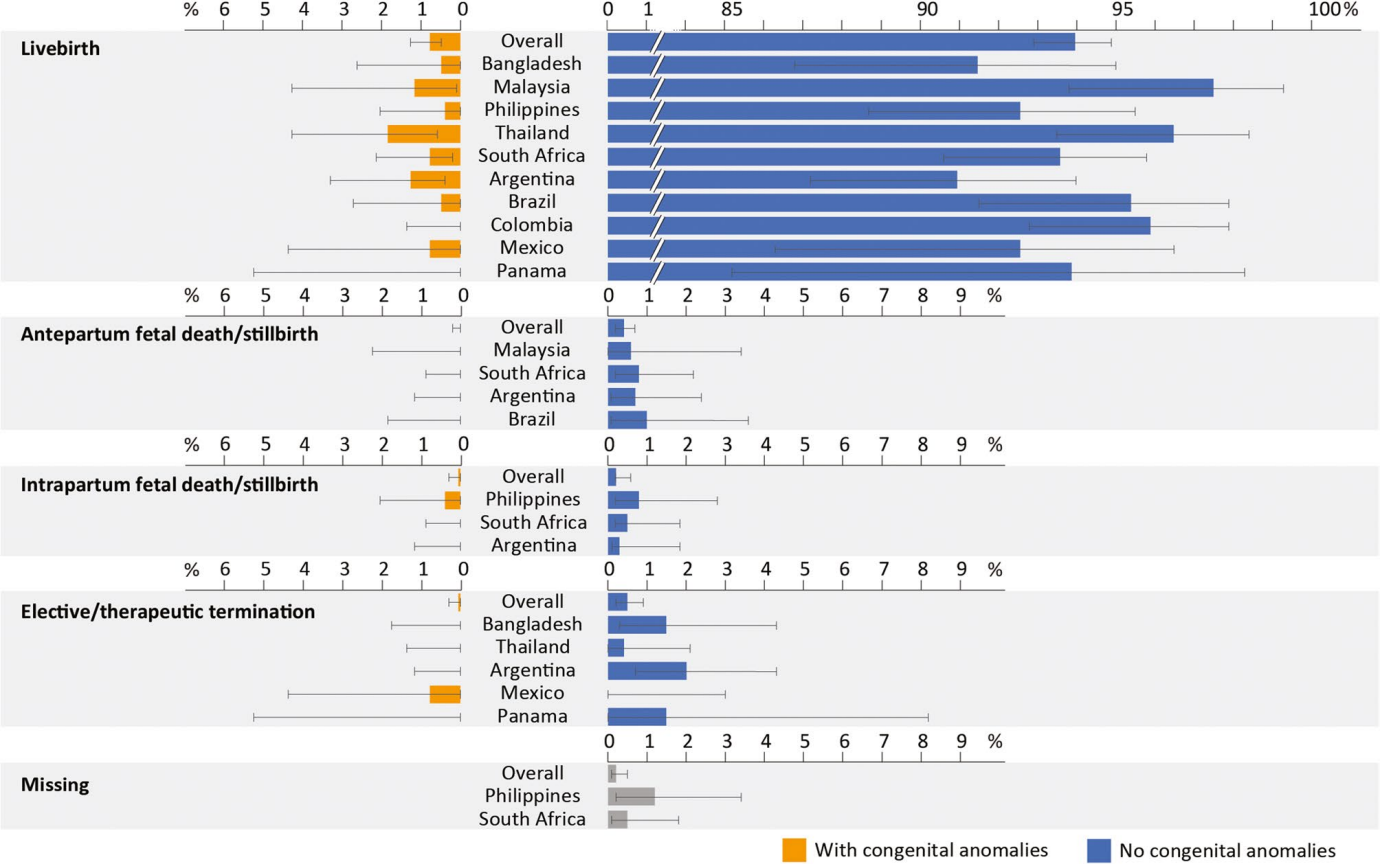
Frequency of pregnancy outcomes, overall and by country (maternal analysis set). Error bars represent 95% confidence intervals Note: Eighty-five (3.8%) participants in the analysis set did not attend the delivery visit and therefore no outcome was reported for their pregnancies

Most pregnancies (2088/2222; 94.0%) resulted in livebirths with no apparent congenital anomalies (ranging from 91.0% in Argentina to 97.5% in Malaysia). Pregnancies resulting in livebirths with apparent congenital anomalies were reported for 18/2022 (0.8%) mothers, with frequencies varying by country from 0% (Colombia and Panama) to 1.9% (Thailand). Antepartum stillbirths without congenital anomalies were reported for 8 (0.4%) pregnant women, from South Africa, Argentina, Brazil, and Malaysia. No case of antepartum fetal death with congenital anomalies was reported. Five (0.2%) pregnant women from the Philippines, South Africa, and Argentina, experienced intrapartum stillbirths without congenital anomalies (Fig. 2, Supplementary Table 5).

Livebirths with no congenital anomaly were reported by 1798 (93.8%) mothers aged 18–34 years, 241 (96.0%) mothers aged 35–39 years, and 49 (90.7%) mothers aged ≥ 40 years.

### Pregnancy-related events of interest

The most frequently reported pregnancy-related EOIs were preterm birth (166/2222; 7.5%), non-reassuring fetal status (NRFS) (137/2222; 6.2%), and hypertensive disorders of pregnancy (125/2222; 5.6%), with the highest reporting rates being observed in South Africa. There was no case of maternal sepsis reported in the study. One maternal death was reported due to postpartum hemorrhage with eclampsia. Other pregnancy-related EOIs considered by the investigators to be of concern were reported for 153 participants (6.9%) (Table 3).

**Table 3:** Percentage of maternal EOIs occurring from first visit to 42 days post-delivery (maternal analysis set)

	% (95% confidence interval)										
Event of interest	Bangladesh (*N* = 201)	Malaysia (*N* = 162)	Philippines (*N* = 258)	Thailand (*N* = 260)	South Africa (*N* = 388)	Argentina (*N* = 301)	Brazil (*N* = 196)	Colombia (*N* = 269)	Mexico (*N* = 121)	Panama (*N* = 66)	Overall (*N* = 2222)
Pathways to preterm birth (at least one event below)	10.4 (6.6, 15.5)	6.8 (3.4, 11.8)	4.3 (2.1, 7.5)	5.0 (2.7, 8.4)	11.3 (8.4, 14.9)	5.0 (2.8, 8.1)	5.6 (2.8, 9.8)	7.1 (4.3, 10.8)	10.7 (5.8, 17.7)	12.1 (5.4, 22.5)	7.5 (6.4, 8.6)
Preterm rupture of membranes	1.5 (0.3, 4.3)	1.9 (0.4, 5.3)	1.2 (0.2, 3.4)	0.0 (0.0, 1.4)	2.3 (1.1, 4.4)	2.3 (0.9, 4.7)	2.0 (0.6, 5.1)	2.6 (1.1, 5.3)	0.8 (0.0, 4.5)	4.5 (0.9, 12.7)	1.8 (1.3, 2.4)
Preterm labor	4.0 (1.7, 7.7)	3.7 (1.4, 7.9)	3.1 (1.3, 6.0)	4.6 (2.4, 7.9)	7.2 (4.8, 10.3)	2.3 (0.9, 4.7)	3.1 (1.1, 6.5)	4.1 (2.1, 7.2)	9.9 (5.2, 16.7)	4.5 (0.9, 12.7)	4.5 (3.7, 5.5)
Provider-initiated preterm birth	5.0 (2.4, 9.0)	1.2 (0.1, 4.4)	0.0 (0.0, 1.4)	0.4 (0.0, 2.1)	1.8 (0.7, 3.7)	0.3 (0.0, 1.8)	0.5 (0.0, 2.8)	0.4 (0.0, 2.1)	0.0 (0.0, 3.0)	3.0 (0.4, 10.5)	1.1 (0.7, 1.7)
NRFS	0.0 (0.0, 1.8)	6.2 (3.0, 11.1)	5.0 (2.7, 8.5)	1.5 (0.4, 3.9)	18.3 (14.6, 22.5)	0.3 (0.0, 1.8)	11.7 (7.6, 17.1)	4.5 (2.3, 7.7)	0.0 (0.0, 3.0)	4.5 (0.9, 12.7)	6.2 (5.2, 7.2)
Hypertensive disorders of pregnancy (at least one event below)	0.5 (0.0, 2.7)	2.5 (0.7, 6.2)	4.7 (2.4, 8.0)	2.7 (1.1, 5.5)	9.8 (7.0, 13.2)	7.3 (4.6, 10.9)	9.2 (5.5, 14.1)	6.3 (3.7, 9.9)	3.3 (0.9, 8.2)	3.0 (0.4, 10.5)	5.6 (4.7, 6.7)
Gestational hypertension	0.5 (0.0, 2.7)	1.9 (0.4, 5.3)	2.3 (0.9, 5.0)	1.2 (0.2, 3.3)	5.9 (3.8, 8.8)	5.0 (2.8, 8.1)	5.6 (2.8, 9.8)	2.6 (1.1, 5.3)	2.5 (0.5, 7.1)	0.0 (0.0, 5.4)	3.2 (2.5, 4.1)
Pre-eclampsia	0.0 (0.0, 1.8)	0.6 (0.0, 3.4)	1.6 (0.4, 3.9)	0.8 (0.1, 2.8)	3.1 (1.6, 5.3)	1.0 (0.2, 2.9)	3.1 (1.1, 6.5)	3.0 (1.3, 5.8)	0.0 (0.0, 3.0)	1.5 (0.0, 8.2)	1.7 (1.2, 2.3)
Pre-eclampsia with severe features (including eclampsia)	0.0 (0.0, 1.8)	0.0 (0.0, 2.3)	0.8 (0.1, 2.8)	0.8 (0.1, 2.8)	0.8 (0.2, 2.2)	1.3 (0.4, 3.4)	0.5 (0.0, 2.8)	0.7 (0.1, 2.7)	0.8 (0.0, 4.5)	1.5 (0.0, 8.2)	0.7 (0.4, 1.2)
Dysfunctional labor^a^ (at least one event below)	1.5 (0.3, 4.3)	0.6 (0.0, 3.4)	4.7 (2.4, 8.0)	4.6 (2.4, 7.9)	4.4 (2.6, 6.9)	1.7 (0.5, 3.8)	6.1 (3.2, 10.5)	5.6 (3.2, 9.0)	0.8 (0.0, 4.5)	3.0 (0.4, 10.5)	3.6 (2.9, 4.5)
First stage of labor	1.5 (0.3, 4.3)	0.0 (0.0, 2.3)	2.3 (0.9, 5.0)	3.1 (1.3, 6.0)	2.3 (1.1, 4.4)	1.3 (0.4, 3.4)	3.6 (1.4, 7.2)	4.1 (2.1, 7.2)	0.0 (0.0, 3.0)	1.5 (0.0, 8.2)	2.2 (1.6, 2.9)
Second stage of labor	0.0 (0.0, 1.8)	0.6 (0.0, 3.4)	2.3 (0.9, 5.0)	1.5 (0.4, 3.9)	2.1 (0.9, 4.0)	0.3 (0.0, 1.8)	2.6 (0.8, 5.9)	1.5 (0.4, 3.8)	0.8 (0.0, 4.5)	1.5 (0.0, 8.2)	1.4 (0.9, 2.0)
Gestational diabetes mellitus	0.0 (0.0, 1.8)	6.2 (3.0, 11.1)	2.3 (0.9, 5.0)	5.4 (3.0, 8.9)	0.8 (0.2, 2.2)	3.3 (1.6, 6.0)	5.6 (2.8, 9.8)	0.7 (0.1, 2.7)	1.7 (0.2, 5.8)	0.0 (0.0, 5.4)	2.6 (2.0, 3.4)
Oligohydramnios	5.0 (2.4, 9.0)	1.2 (0.1, 4.4)	1.2 (0.2, 3.4)	1.2 (0.2, 3.3)	0.5 (0.1, 1.8)	1.3 (0.4, 3.4)	3.1 (1.1, 6.5)	0.4 (0.0, 2.1)	8.3 (4.0, 14.7)	1.5 (0.0, 8.2)	1.9 (1.4, 2.5)
Postpartum hemorrhage	0.5 (0.0, 2.7)	4.9 (2.2, 9.5)	0.4 (0.0, 2.1)	0.8 (0.1, 2.8)	3.4 (1.8, 5.7)	0.7 (0.1, 2.4)	2.0 (0.6, 5.1)	1.1 (0.2, 3.2)	2.5 (0.5, 7.1)	0.0 (0.0, 5.4)	1.7 (1.2, 2.3)
Fetal growth restriction	0.5 (0.0, 2.7)	0.6 (0.0, 3.4)	0.0 (0.0, 1.4)	0.8 (0.1, 2.8)	0.5 (0.1, 1.8)	0.7 (0.1, 2.4)	2.6 (0.8, 5.9)	1.9 (0.6, 4.3)	0.8 (0.0, 4.5)	0.0 (0.0, 5.4)	0.9 (0.5, 1.3)
Chorioamnionitis	0.0 (0.0, 1.8)	1.2 (0.1, 4.4)	0.4 (0.0, 2.1)	0.0 (0.0, 1.4)	0.3 (0.0, 1.4)	0.0 (0.0, 1.2)	2.0 (0.6, 5.1)	1.5 (0.4, 3.8)	0.8 (0.0, 4.5)	0.0 (0.0, 5.4)	0.6 (0.3, 1.0)
Antenatal bleeding (at least one event below)	0.0 (0.0, 1.8)	0.6 (0.0, 3.4)	0.0 (0.0, 1.4)	0.0 (0.0, 1.4)	1.3 (0.4, 3.0)	1.0 (0.2, 2.9)	0.0 (0.0, 1.9)	0.7 (0.1, 2.7)	0.0 (0.0, 3.0)	0.0 (0.0, 5.4)	0.5 (0.2, 0.9)
Placenta accreta spectrum disorders	0.0 (0.0, 1.8)	0.0 (0.0, 2.3)	0.0 (0.0, 1.4)	0.0 (0.0, 1.4)	0.0 (0.0, 0.9)	0.0 (0.0, 1.2)	0.0 (0.0, 1.9)	0.4 (0.0, 2.1)	0.0 (0.0, 3.0)	0.0 (0.0, 5.4)	0.1 (0.0, 0.3)
Placental abruption	0.0 (0.0, 1.8)	0.6 (0.0, 3.4)	0.0 (0.0, 1.4)	0.0 (0.0, 1.4)	1.3 (0.4, 3.0)	1.0 (0.2, 2.9)	0.0 (0.0, 1.9)	0.4 (0.0, 2.1)	0.0 (0.0, 3.0)	0.0 (0.0, 5.4)	0.5 (0.2, 0.8)
Cesarean scar pregnancy^b^	0.0 (0.0, 1.8)	0.0 (0.0, 2.3)	0.0 (0.0, 1.4)	0.0 (0.0, 1.4)	0.0 (0.0, 0.9)	0.0 (0.0, 1.2)	0.0 (0.0, 1.9)	0.0 (0.0, 1.4)	0.0 (0.0, 3.0)	0.0 (0.0, 5.4)	0.0 (0.0, 0.2)
Uterine rupture	0.0 (0.0, 1.8)	0.0 (0.0, 2.3)	0.0 (0.0, 1.4)	0.0 (0.0, 1.4)	0.0 (0.0, 0.9)	0.0 (0.0, 1.2)	0.0 (0.0, 1.9)	0.0 (0.0, 1.4)	0.0 (0.0, 3.0)	0.0 (0.0, 5.4)	0.0 (0.0, 0.2)
Polyhydramnios	0.0 (0.0, 1.8)	1.2 (0.1, 4.4)	0.4 (0.0, 2.1)	0.4 (0.0, 2.1)	0.0 (0.0, 0.9)	0.0 (0.0, 1.2)	1.5 (0.3, 4.4)	0.4 (0.0, 2.1)	0.0 (0.0, 3.0)	1.5 (0.0, 8.2)	0.4 (0.2, 0.8)
Gestational liver disease (at least one event below)	0.0 (0.0, 1.8)	0.0 (0.0, 2.3)	0.0 (0.0, 1.4)	0.0 (0.0, 1.4)	0.0 (0.0, 0.9)	0.3 (0.0, 1.8)	0.0 (0.0, 1.9)	0.4 (0.0, 2.1)	0.8 (0.0, 4.5)	0.0 (0.0, 5.4)	0.1 (0.0, 0.4)
Intrahepatic cholestasis of pregnancy	0.0 (0.0, 1.8)	0.0 (0.0, 2.3)	0.0 (0.0, 1.4)	0.0 (0.0, 1.4)	0.0 (0.0, 0.9)	0.3 (0.0, 1.8)	0.0 (0.0, 1.9)	0.4 (0.0, 2.1)	0.8 (0.0, 4.5)	0.0 (0.0, 5.4)	0.1 (0.0, 0.4)
Acute fatty liver of pregnancy	0.0 (0.0, 1.8)	0.0 (0.0, 2.3)	0.0 (0.0, 1.4)	0.0 (0.0, 1.4)	0.0 (0.0, 0.9)	0.0 (0.0, 1.2)	0.0 (0.0, 1.9)	0.0 (0.0, 1.4)	0.0 (0.0, 3.0)	0.0 (0.0, 5.4)	0.0 (0.0, 0.2)
Maternal death	0.5 (0.0, 2.7)	0.0 (0.0, 2.3)	0.0 (0.0, 1.4)	0.0 (0.0, 1.4)	0.0 (0.0, 0.9)	0.0 (0.0, 1.2)	0.0 (0.0, 1.9)	0.0 (0.0, 1.4)	0.0 (0.0, 3.0)	0.0 (0.0, 5.4)	0.1 (0.0, 0.3)
Maternal sepsis^c^	0.0 (0.0, 1.8)	0.0 (0.0, 2.3)	0.0 (0.0, 1.4)	0.0 (0.0, 1.4)	0.0 (0.0, 0.9)	0.0 (0.0, 1.2)	0.0 (0.0, 1.9)	0.0 (0.0, 1.4)	0.0 (0.0, 3.0)	0.0 (0.0, 5.4)	0.0 (0.0, 0.2)
Other^d^	10.9 (7.0, 16.1)	10.5 (6.2, 16.3)	4.3 (2.1, 7.5)	1.5 (0.4, 3.9)	13.9 (10.6, 17.8)	3.3 (1.6, 6.0)	10.7 (6.8, 15.9)	2.6 (1.1, 5.3)	0.8 (0.0, 4.5)	9.1 (3.4, 18.7)	6.9 (5.9, 8.0)
Missing	0.0 (0.0, 1.8)	0.0 (0.0, 2.3)	0.0 (0.0, 1.4)	0.0 (0.0, 1.4)	0.0 (0.0, 0.9)	0.0 (0.0, 1.2)	0.0 (0.0, 1.9)	0.4 (0.0, 2.1)	0.0 (0.0, 3.0)	0.0 (0.0, 5.4)	0.1 (0.0, 0.3)

*EOI* event of interest, *%* percentage of participants with the event of interest, *N* number of participants included in the analyses, *NRFS* non-reassuring fetal status

^a^Dysfunctional labor is defined as prolonged labor at or after 37 weeks and before 42 weeks of gestational age in singleton pregnancies. First and second stage of labor are defined according to the Global Alignment of Immunization safety Assessment (GAIA) case definition [40]

^b^During the conduct of the study, the term “cesarean scar pregnancy” was used to define all hysterotomy scar pregnancies, being considered a source for antenatal bleeding [43]. In 2018, approximatively one year after the protocol was finalized, the International Federation of Gynecology and Obstetrics consensus guidelines recommended the use of the terminology “placenta accreta spectrum disorders” in epidemiologic studies reporting on adherent and invasive placental disorders, which are most likely preceded by a cesarean scar pregnancy [51, 52]. Conditions like “cesarean scar pregnancy” and “placenta accreta spectrum disorders” share common histological features and represent a continuum of the same disease [53]. Therefore, the terms “cesarean scar pregnancy” (diagnosed in the first and second trimester of pregnancy) and “placenta accreta spectrum disorders” (diagnosed in the second trimester and beyond) can be considered in future studies to describe different stages of the same disease

^c^Maternal sepsis is defined as organ dysfunction resulting from infection during pregnancy, childbirth, post-abortion, or post-partum period. A detailed definition of maternal sepsis is provided in Supplementary Table 3

^d^Any other pregnancy-related event considered by the investigator to be of concern

When classified according to GAIA levels of diagnostic certainty (Supplementary Table 6), preterm labor and preterm rupture of membranes events were distributed across all levels of diagnostic certainty. Most provider-initiated preterm births (23/25) corresponded to level 1 GAIA definition while NRFS cases were distributed across all three levels of GAIA diagnostic certainty. Most gestational hypertension cases (50/72) were classified as GAIA level 1 and 2, while most first stage and second stage dysfunctional labor cases (51/80) met the GAIA level 1 definition. The only case of maternal death met the GAIA level 3 definition.

### Neonatal events of interest

The most frequently reported EOIs in neonates were low birthweight (LBW [< 2500 g], including very LBW [< 1500 g]; 156/2094; 7.4%), preterm birth (141/2094; 6.7%), and small for gestational age (SGA; 111/2094; 5.3%). Congenital anomalies were reported for 103/2094 (4.9%) neonates: major external structural defects (38/2094; 1.8%), internal structural defects (46/2094; 2.2%), and functional defects (19/2094; 0.9%); percentages varied by country (Table 4).

**Table 4:** Percentage of neonatal EOIs occurring from birth to 28 days post-delivery (neonatal analysis set)

	% (95% confidence interval)										
Event of interest	Bangladesh (*N* = 190)	Malaysia (*N* = 159)	Philippines (*N* = 240)	Thailand (*N* = 255)	South Africa (*N* = 373)	Argentina (*N* = 283)	Brazil (*N* = 183)	Colombia (*N* = 255)	Mexico (*N* = 95)	Panama (*N* = 61)	Overall (*N* = 2094)
LBW including very LBW	12.1 (7.8, 17.6)	11.9 (7.4, 18.0)	12.9 (8.9, 17.8)	4.3 (2.2, 7.6)	8.3 (5.7, 11.6)	4.9 (2.7, 8.2)	3.8 (1.6, 7.7)	4.3 (2.2, 7.6)	6.3 (2.4, 13.2)	4.9 (1.0, 13.7)	7.4 (6.4, 8.7)
Preterm birth	12.6 (8.3, 18.2)	8.2 (4.4, 13.6)	4.6 (2.3, 8.1)	5.5 (3.0, 9.0)	8.3 (5.7, 11.6)	5.7 (3.3, 9.0)	4.9 (2.3, 9.1)	5.1 (2.7, 8.6)	5.3 (1.7, 11.9)	8.2 (2.7, 18.1)	6.7 (5.7, 7.9)
SGA	11.6 (7.4, 17.0)	6.3 (3.1, 11.3)	6.3 (3.5, 10.1)	3.1 (1.4, 6.1)	7.8 (5.3, 11.0)	2.5 (1.0, 5.0)	4.4 (1.9, 8.4)	3.1 (1.4, 6.1)	4.2 (1.2, 10.4)	0.0 (0.0, 5.9)	5.3 (4.4, 6.3)
Congenital anomalies (at least one event below)	1.6 (0.3, 4.5)	3.8 (1.4, 8.0)	1.7 (0.5, 4.2)	3.9 (1.9, 7.1)	13.7 (10.4, 17.6)	3.9 (2.0, 6.8)	3.8 (1.6, 7.7)	1.2 (0.2, 3.4)	8.4 (3.7, 15.9)	0.0 (0.0, 5.9)	4.9 (4.0, 5.9)
Major external structural defects	0.5 (0.0, 2.9)	0.0 (0.0, 2.3)	0.0 (0.0, 1.5)	0.8 (0.1, 2.8)	7.8 (5.3, 11.0)	0.4 (0.0, 2.0)	1.1 (0.1, 3.9)	0.4 (0.0, 2.2)	2.1 (0.3, 7.4)	0.0 (0.0, 5.9)	1.8 (1.3, 2.5)
Internal structural defects	0.5 (0.0, 2.9)	1.3 (0.2, 4.5)	1.3 (0.3, 3.6)	0.0 (0.0, 1.4)	5.4 (3.3, 8.2)	2.8 (1.2, 5.5)	2.2 (0.6, 5.5)	0.8 (0.1, 2.8)	6.3 (2.4, 13.2)	0.0 (0.0, 5.9)	2.2 (1.6, 2.9)
Functional defects	0.5 (0.0, 2.9)	2.5 (0.7, 6.3)	0.4 (0.0, 2.3)	3.1 (1.4, 6.1)	0.5 (0.1, 1.9)	0.7 (0.1, 2.5)	0.5 (0.0, 3.0)	0.0 (0.0, 1.4)	0.0 (0.0, 3.8)	0.0 (0.0, 5.9)	0.9 (0.5, 1.4)
Respiratory distress in the neonate	1.6 (0.3, 4.5)	4.4 (1.8, 8.9)	2.5 (0.9, 5.4)	2.4 (0.9, 5.1)	5.9 (3.7, 8.8)	1.8 (0.6, 4.1)	12.0 (7.7, 17.6)	5.5 (3.0, 9.0)	7.4 (3.0, 14.6)	3.3 (0.4, 11.3)	4.5 (3.6, 5.5)
Neonatal infections (at least one event below)	6.3 (3.3, 10.8)	1.3 (0.2, 4.5)	3.3 (1.4, 6.5)	1.6 (0.4, 4.0)	5.4 (3.3, 8.2)	0.0 (0.0, 1.3)	6.0 (3.0, 10.5)	2.4 (0.9, 5.1)	1.1 (0.0, 5.7)	8.2 (2.7, 18.1)	3.3 (2.6, 4.2)
Blood stream infections	1.6 (0.3, 4.5)	0.6 (0.0, 3.5)	1.7 (0.5, 4.2)	0.4 (0.0, 2.2)	2.4 (1.1, 4.5)	0.0 (0.0, 1.3)	4.4 (1.9, 8.4)	0.8 (0.1, 2.8)	1.1 (0.0, 5.7)	6.6 (1.8, 15.9)	1.6 (1.1, 2.2)
Meningitis	0.0 (0.0, 1.9)	0.0 (0.0, 2.3)	0.0 (0.0, 1.5)	0.0 (0.0, 1.4)	0.0 (0.0, 1.0)	0.0 (0.0, 1.3)	0.0 (0.0, 2.0)	0.0 (0.0, 1.4)	0.0 (0.0, 3.8)	0.0 (0.0, 5.9)	0.0 (0.0, 0.2)
Respiratory infection	4.7 (2.2, 8.8)	0.6 (0.0, 3.5)	1.7 (0.5, 4.2)	1.2 (0.2, 3.4)	2.9 (1.5, 5.2)	0.0 (0.0, 1.3)	1.6 (0.3, 4.7)	1.6 (0.4, 4.0)	0.0 (0.0, 3.8)	1.6 (0.0, 8.8)	1.7 (1.2, 2.4)
Large for gestational age	0.0 (0.0, 1.9)	1.9 (0.4, 5.4)	2.1 (0.7, 4.8)	3.9 (1.9, 7.1)	2.7 (1.3, 4.9)	1.4 (0.4, 3.6)	8.2 (4.7, 13.2)	1.2 (0.2, 3.4)	3.2 (0.7, 9.0)	1.6 (0.0, 8.8)	2.6 (1.9, 3.4)
Macrosomia	0.0 (0.0, 1.9)	0.6 (0.0, 3.5)	1.3 (0.3, 3.6)	0.4 (0.0, 2.2)	4.0 (2.3, 6.5)	3.9 (2.0, 6.8)	8.2 (4.7, 13.2)	1.2 (0.2, 3.4)	3.2 (0.7, 9.0)	1.6 (0.0, 8.8)	2.5 (1.9, 3.3)
Congenital microcephaly (at least one event below)	0.0 (0.0, 1.9)	0.0 (0.0, 2.3)	0.0 (0.0, 1.5)	1.2 (0.2, 3.4)	3.5 (1.9, 5.9)	0.0 (0.0, 1.3)	0.0 (0.0, 2.0)	0.0 (0.0, 1.4)	1.1 (0.0, 5.7)	0.0 (0.0, 5.9)	0.8 (0.5, 1.3)
Postnatally diagnosed	0.0 (0.0, 1.9)	0.0 (0.0, 2.3)	0.0 (0.0, 1.5)	0.8 (0.1, 2.8)	3.5 (1.9, 5.9)	0.0 (0.0, 1.3)	0.0 (0.0, 2.0)	0.0 (0.0, 1.4)	1.1 (0.0, 5.7)	0.0 (0.0, 5.9)	0.8 (0.4, 1.2)
Prenatally diagnosed	0.0 (0.0, 1.9)	0.0 (0.0, 2.3)	0.0 (0.0, 1.5)	0.4 (0.0, 2.2)	0.0 (0.0, 1.0)	0.0 (0.0, 1.3)	0.0 (0.0, 2.0)	0.0 (0.0, 1.4)	0.0 (0.0, 3.8)	0.0 (0.0, 5.9)	0.05 (0.0, 0.3)
Neonatal death (at least one event below)	1.6 (0.3, 4.5)	0.0 (0.0, 2.3)	0.8 (0.1, 3.0)	0.0 (0.0, 1.4)	1.1 (0.3, 2.7)	0.0 (0.0, 1.3)	0.0 (0.0, 2.0)	0.0 (0.0, 1.4)	1.1 (0.0, 5.7)	0.0 (0.0, 5.9)	0.5 (0.2, 0.9)
Neonatal death in a preterm livebirth	0.5 (0.0, 2.9)	0.0 (0.0, 2.3)	0.4 (0.0, 2.3)	0.0 (0.0, 1.4)	0.3 (0.0, 1.5)	0.0 (0.0, 1.3)	0.0 (0.0, 2.0)	0.0 (0.0, 1.4)	0.0 (0.0, 3.8)	0.0 (0.0, 5.9)	0.1 (0.0, 0.4)
Neonatal death in a term livebirth	1.1 (0.1, 3.8)	0.0 (0.0, 2.3)	0.4 (0.0, 2.3)	0.0 (0.0, 1.4)	0.8 (0.2, 2.3)	0.0 (0.0, 1.3)	0.0 (0.0, 2.0)	0.0 (0.0, 1.4)	1.1 (0.0, 5.7)	0.0 (0.0, 5.9)	0.3 (0.1, 0.7)
Neonatal encephalopathy	0.0 (0.0, 1.9)	0.6 (0.0, 3.5)	0.4 (0.0, 2.3)	0.0 (0.0, 1.4)	0.3 (0.0, 1.5)	0.4 (0.0, 2.0)	0.0 (0.0, 2.0)	0.8 (0.1, 2.8)	0.0 (0.0, 3.8)	0.0 (0.0, 5.9)	0.3 (0.1, 0.6)
Failure to thrive	0.5 (0.0, 2.9)	0.6 (0.0, 3.5)	0.8 (0.1, 3.0)	0.4 (0.0, 2.2)	0.5 (0.1, 1.9)	0.0 (0.0, 1.3)	0.0 (0.0, 2.0)	0.0 (0.0, 1.4)	0.0 (0.0, 3.8)	0.0 (0.0, 5.9)	0.3 (0.1, 0.7)
Other^a^	14.2 (9.6, 20.0)	5.0 (2.2, 9.7)	3.8 (1.7, 7.0)	22.0 (17.0, 27.5)	11.0 (8.0, 14.6)	1.4 (0.4, 3.6)	19.1 (13.7, 25.6)	5.5 (3.0, 9.0)	7.4 (3.0, 14.6)	19.7 (10.6, 31.8)	10.2 (8.9, 11.5)

*EOI* event of interest, *%* percentage of participants with the event of interest, *N* number of participants included in the analyses, *SGA* small for gestational age, *LBW* low birthweight

Note: ^a^Any other neonatal event considered by the investigator to be of concern

Death occurred in 10 (0.5%) neonates, 3 (1.6%) in Bangladesh, 4 (1.1%) in South Africa, 1 (1.1%) in Mexico, and 2 (0.8%) in the Philippines. No deaths were reported in the other countries. Seven neonatal deaths occurred in full-term livebirths and were due to congenital heart disease/neonatal sepsis, meconium aspiration, pulmonary atresia, respiratory distress (for two neonates), sepsis with pulmonary hemorrhage, and aspiration during feeding. The three neonatal deaths in preterm births were caused by neonatal pneumonia, respiratory distress, and blood stream infection. A total of 213 (10.2%) other neonatal EOIs were considered by the investigator to be of concern (Table 4), of which, the most frequently reported included tongue tie (*n* = 44), umbilical hernia (*n* = 27), and neonatal jaundice (*n* = 23).

Eighty-seven events (4.8%) of congenital anomalies were reported in the maternal age group of 18–34 years and 16 events (6.6%) in the maternal age group of 35–39 years. No event of congenital anomaly occurred in neonates of mothers in the age group of ≥ 40 years.

When classified according to GAIA levels of diagnostic certainty, most LBW events (106/156; 67.9%) and preterm births (73/141; 51.8%) met the GAIA level 1 definition. SGA cases were more evenly distributed across the first two levels of diagnostic certainty (Supplementary Table 7).

### Risk factors for maternal and neonatal events of interest

In univariate logistic regression analyses evaluating the association between pregnancy-related EOIs and baseline characteristics, several variables had p-values ≤ 0.1 and were selected for multivariate logistic regression analyses (Supplementary Table 8). Most of these variables reached statistically significant p-values of ≤ 0.05. For instance, each one-unit increase in the pre-pregnancy BMI was shown to increase the odds of having hypertensive disorders of pregnancy by 1.16 times (odds ratio [OR] = 1.16 [95% CI 1.1, 1.22]), gestational hypertension by 1.17 times (OR = 1.17 [95% CI 1.09, 1.26]), pre-eclampsia by 1.1 times (OR = 1.1 [95% CI 1.03, 1.18]), dysfunctional labor by 1.06 times (OR = 1.06 [95% CI 1.01, 1.11]), and NRFS by 1.07 times (OR = 1.07 [95% CI 1.03, 1.11]). The odds of gestational diabetes mellitus were shown to be increased by a high education level (Bachelor’s degree or higher) of the mother (OR = 2.29 [95% CI 1.05, 5.01]) or by a cesarean section in previous pregnancy (OR = 2.35 [95% CI 1.13, 4.89]) (Supplementary Table 9).

Of the variables selected from univariate logistic regression testing (Supplementary Table 10), prenatal smoking exposure, high maternal pre-pregnancy BMI, maternal gestational hypertension during pregnancy, fetal growth restriction, and female sex of the neonate still showed a trend for association with neonatal EOIs in multivariate logistic regression analyses. For instance, the odds of having congenital anomalies were shown to increase by 4.3 times with prenatal smoking exposure (OR = 4.3 [95% CI 2.55, 7.25]) and by 6.3 times with gestational hypertension (OR = 6.32 [95% CI 1.86, 21.47]) (Supplementary Table 11).

## Discussion

In this study, we assessed the rates of pregnancy outcomes, pregnancy-related and neonatal EOIs in relatively healthy mothers and their neonates. Overall, we observed a similar frequency in pregnancy outcomes, pregnancy-related and neonatal EOIs across countries, although some variation was noted.

Livebirths with apparent congenital anomalies were reported for 0.8% pregnancy outcomes in our study and 4.9% of neonates had congenital anomalies, although this rate varied by country. This apparent discrepancy can be explained by the fact that major congenital anomalies were generally detected by ultrasonic scan at 18–20 weeks gestational age and were excluded if being detected. In contrast, the minor ones might have been easily missed by the anomaly scan, being identifiable during the additional clinical evaluation in the neonates. As such, congenital anomalies reported in our study did not lead to a high neonatal mortality: a rate of 0.5% was observed, while a rate of 18 deaths/1000 livebirths was reported globally in 2021 [54]. Comparison with rates of congenital anomalies reported in other studies is hindered by important differences in methodology (including the GAIA ascertainment and classification of birth defects), and a different period for detecting birth defect (e.g., the entire pregnancy duration versus after gestation week 27 in our study).

The most frequently reported pregnancy-related EOI in our study was preterm delivery (7.5%), followed by NRFS and hypertensive disorders of pregnancy. Preterm birth rates are known to be higher in low-income settings: an average of 12% compared to 9.3% in high-income countries, but large variations are observed among geographic regions [55]. India, Pakistan, Nigeria, China, and Ethiopia accounted for nearly one-half of preterm births that occurred worldwide in 2020 [56]. In 2020, Bangladesh had the highest national rate of preterm births (16.2%) [56], similar to the trend observed in our study. Low household income, low maternal educational status, low BMI, younger maternal age, living in a rural area, and first pregnancy were previously found to be associated with increased chances of preterm birth in African countries [57]. For NRFS, we observed a higher rate in the current study (6.2%), compared to that from a recent cohort study in Japan (3.9%) [58]. However, the data on NRFS remains scarce even for developed countries. The pooled prevalence of hypertensive disorders of pregnancy was estimated at 10% in African countries [59], while estimates in higher-income countries vary from 5.2 to 8.2% [60], compared to the rate of 5.6% observed in our study.

LBW (in 7.4% of neonates) and SGA (5.3%) were among the frequently reported neonatal EOIs in our study. In 2015, the global LBW prevalence was 14.6% [61], but prevalence in LMICs is believed to be underestimated, mainly because neonates are often not weighed after delivery [62] and gestational age is not routinely recorded [24]. SGA rates in our study were the highest in Bangladesh (11.6% of neonates). In 2010, prevalence of SGA was estimated at 5.3–41.5% in LMICs [63], but such assessments are known to be impacted by the reference population used [36].

The absence of standardized case definitions hinders the comparison of safety data across clinical studies and countries [48]. In LMICs, data on maternal and neonatal outcomes is particularly limited and the diagnosis of such events is unharmonized [21]. We used GAIA case definitions/diagnostic levels to standardize our findings and assess the feasibility of applying these definitions in LMICs. Most EOIs in our study were classified as level 1–3 of GAIA diagnostic certainty. However, despite an overall adequate use of case definitions, the diagnosis level was sometimes incorrect or missing, and sometimes a higher classification was not achieved, even when reassessment was attempted with the study sites. Similar challenges in applying the GAIA case definitions were previously observed and discussed for both prospective [50] and retrospective studies [64], emphasizing the need for improved quality of medical records.

This study has several strengths. To date, this is one of the largest prospective studies evaluating the frequency of low-risk pregnancy outcomes, as well as maternal and neonatal EOIs in LMICs, using standardized case definitions. Due to this approach and the level of detail collected for each EOI, our results complement the other available, nationally representative datasets such as those from the Demographic and Health Surveys Program [65]. Despite the serious consequences of COVID-19, leading to significantly increased rates for stillbirth, maternal death, ruptured ectopic pregnancy, preterm birth, and maternal depression [66, 67] there was no apparent increase in the risk of complications due to the disease, and all suspected COVID-19 cases were monitored; only six confirmed cases occurred in the maternal study population and 16 confirmed cases occurred in the infant study population.

The study has several potential limitations. First, all analyses were descriptive, and results should be interpreted with caution considering that there is no adjustment to any potential confounders. Second, relatively healthy women living mostly in urban areas, who had no significant findings observed during the fetal morphology assessment at 24^0/7^–27^6/7^ weeks gestational age, were enrolled and therefore our results cannot be easily generalized to the overall population. Third, in this study we only considered pregnancy-related outcomes and maternal EOIs that might occur after 24 weeks of gestation, when fetuses were viable in most LMICs. Adverse pregnancy outcomes that resulted in the termination of pregnancies before this gestational age could not be demonstrated. We also acknowledge that the likelihood of recording rare and very rare EOIs as part of the study was limited by the relatively low sample size for such events. Furthermore, although GAIA case definitions are useful to adequately define an outcome with different level of certainty, the possibility to use it broadly and as part of standard of care is less certain and it is expected that some sites might have failed to comprehensively classify the EOI based on GAIA case definitions. Considering the limited number of pregnancies in each country, the all-inclusive interpretation of the present study should be done with caution. In addition, the descriptive analysis on risk factors should be interpreted with caution due to lack of multivariable adjustment and restricted selection of the study population (i.e., enrollment of healthy individuals with low-risk pregnancies). Finally, conducting the study during the COVID-19 pandemic has negatively impacted the enrollment of pregnant women and their neonates. All efforts were made to support the countries where the enrollment was hampered by COVID-19; however, we cannot assess the indirect impact the pandemic may have had on the results of this study.

## Conclusions

In conclusion, our study showed that almost all livebirths from LMICs were without apparent congenital anomalies, with preterm delivery and low birthweight being reported in approximatively 7% of pregnant mothers and their neonates, respectively. This study contributed to establishing most recent background rates for pregnancy outcomes, as well as maternal and neonatal EOIs in LMICs’ low-risk pregnant women, where knowledge gaps were frequently observed. This study is expected to facilitate the assessment and interpretation of safety data (including the investigation of safety signals, or evaluation of causal assessments following immunization), in future interventional trials in pregnant women and neonates from LMICs.

## Supplementary Information

Supplementary Material 1.

## Data Availability

Study data and documents can be requested for further research from www.clinicalstudydatarequest.com.

## Abbreviations

BMI: body mass index
CI: confidence interval
EOI: event of interest
GAIA: Global Alignment of Immunization safety Assessment
LBW: low birthweight
LMIC: low- and middle-income country
NRFS: non-reassuring fetal status
OR: odds ratio
RSV: respiratory syncytial virus
SGA: small for gestational age

## References

[1] Kourtis AP, Read JS, Jamieson DJ. Pregnancy and infection. N Engl J Med. 2014;370(23):2211–8. 24897084 10.1056/NEJMra1213566 PMC4459512

[2] Albrecht M, Arck PC. Vertically transferred immunity in neonates: mothers, mechanisms and mediators. Front Immunol. 2020;11: 555. 32296443 10.3389/fimmu.2020.00555 PMC7136470

[3] Marshall H, McMillan M, Andrews RM, Macartney K, Edwards K. Vaccines in pregnancy: the dual benefit for pregnant women and infants. Hum Vaccin Immunother. 2016;12(4):848–56. 26857450 10.1080/21645515.2015.1127485 PMC4962964

[4] Löwensteyn YN, Nair H, Nunes MC, van Roessel I, Vernooij FS, Willemsen J, et al. Estimated impact of maternal vaccination on global paediatric influenza-related in-hospital mortality: a retrospective case series. EClinicalMedicine. 2021;37: 100945. 34386739 10.1016/j.eclinm.2021.100945 PMC8343247

[5] ACOG Committee Opinion 741. Maternal immunization. Obstet Gynecol. 2018;131(6):e214–7. 29794683 10.1097/AOG.0000000000002662

[6] World Health Organization. Questions and answers: COVID-19 vaccines and pregnancy. https://www.who.int/publications/i/item/WHO-2019-nCoV-FAQ-Pregnancy-Vaccines-2022.1 (2022). Accessed 6 Jan 2023.

[7] Centers for Disease Control and Prevention. COVID-19 vaccines while pregnant or breastfeeding. https://www.cdc.gov/coronavirus/2019-ncov/vaccines/recommendations/pregnancy.html (2022). Accessed 11 Nov 2022.

[8] National Health Service (NHS). Vaccinations in pregnancy. https://www.nhs.uk/pregnancy/keeping-well/vaccinations/ (2019). Accessed 11 Nov 2022.

[9] World Health Organization. Table 1: summary of WHO position papers - recommendations for routine immunization. https://www.who.int/teams/immunization-vaccines-and-biologicals/policies/who-recommendations-for-routine-immunization---summary-tables (2023). Accessed 6 Jan 2023.

[10] Engmann C, Fleming JA, Khan S, Innis BL, Smith JM, Hombach J, et al. Closer and closer? Maternal immunization: current promise, future horizons. J Perinatol. 2020;40(6):844–57. 32341454 10.1038/s41372-020-0668-3 PMC7223555

[11] Rasmussen SA, Watson AK, Kennedy ED, Broder KR, Jamieson DJ. Vaccines and pregnancy: past, present, and future. Semin Fetal Neonatal Med. 2014;19(3):161–9. 24355683 10.1016/j.siny.2013.11.014

[12] Vojtek I, Dieussaert I, Doherty TM, Franck V, Hanssens L, Miller J, et al. Maternal immunization: where are we now and how to move forward? Ann Med. 2018;50(3):193–208. 29308916 10.1080/07853890.2017.1421320

[13] Etti M, Calvert A, Galiza E, Lim S, Khalil A, Le Doare K, et al. Maternal vaccination: a review of current evidence and recommendations. Am J Obstet Gynecol. 2022;226(4):459–74. 34774821 10.1016/j.ajog.2021.10.041 PMC8582099

[14] Regan AK, de Klerk N, Moore HC, Omer SB, Shellam G, Effler PV. Effect of maternal influenza vaccination on hospitalization for respiratory infections in newborns: a retrospective cohort study. Pediatr Infect Dis J. 2016;35(10):1097–103. 27314823 10.1097/INF.0000000000001258

[15] Omer SB. Maternal immunization. N Engl J Med. 2017;376(13):1256–67. 28355514 10.1056/NEJMra1509044

[16] Sharan A, Stuurman AL, Jahagirdar S, Elango V, Riera-Montes M, Kashyap NK, et al. Estimating baseline rates of adverse perinatal and neonatal outcomes using a facility-based surveillance approach: a prospective observational study from the WHO global vaccine safety multi-country collaboration on safety in pregnancy. E Clin Med. 2022;50: 101506. 10.1016/j.eclinm.2022.101506 PMC9234094 35770255

[17] Sedgh G, Singh S, Hussain R. Intended and unintended pregnancies worldwide in 2012 and recent trends. Stud Fam Plann. 2014;45(3):301–14. 25207494 10.1111/j.1728-4465.2014.00393.x PMC4727534

[18] Smid MC, Stringer EM, Stringer JSA. A worldwide epidemic: the problem and challenges of preterm birth in low- and middle-income countries. Am J Perinatol. 2016;33(3):276–89. 26841086 10.1055/s-0035-1571199

[19] UNICEF. Antenatal care. https://data.unicef.org/topic/maternal-health/antenatal-care/ (2021). Accessed 27 Jan 2023.

[20] McDonald CR, Weckman AM, Wright JK, Conroy AL, Kain KC. Pregnant women in low- and middle-income countries require a special focus during the COVID-19 pandemic. Front Glob Womens Health. 2020;1:564560. 34816152 10.3389/fgwh.2020.564560 PMC8594030

[21] Kochhar S, Bonhoeffer J, Jones CE, Muñoz FM, Honrado A, Bauwens J, et al. Immunization in pregnancy clinical research in low- and middle-income countries - study design, regulatory and safety considerations. Vaccine. 2017;35(48 Pt A):6575–81. 28479177 10.1016/j.vaccine.2017.03.103 PMC5714435

[22] Dieussaert I, Hyung Kim J, Luik S, Seidl C, Pu W, Stegmann J-U, et al. RSV prefusion F protein–based maternal vaccine — preterm birth and other outcomes. N Engl J Med. 2024;390(11):1009–21. 38477988 10.1056/NEJMoa2305478

[23] The World Bank Group. World Bank country and lending groups: historical classification by income. https://datahelpdesk.worldbank.org/knowledgebase/articles/906519. Accessed 16 May 2023.

[24] Quinn JA, Munoz FM, Gonik B, Frau L, Cutland C, Mallett-Moore T, et al. Preterm birth: case definition & guidelines for data collection, analysis, and presentation of immunisation safety data. Vaccine. 2016;34(49):6047–56. 27743648 10.1016/j.vaccine.2016.03.045 PMC5139808

[25] UCSF ObGyn&RS Maternal-Fetal Medicine &. Reproductive Genetics: Low-Risk Pregnancies. https://obgyn.ucsf.edu/maternal-fetal-medicine/low-risk-pregnancies#:~:text=The%20majority%20of%20pregnancies%20are,at%20increased%20risk%20for%20complications. Accessed 16 May 2023.

[26] World Health Organization. WHO technical consultation on postpartum and postnatal care: Chap. 6. https://www.ncbi.nlm.nih.gov/books/NBK310591/pdf/Bookshelf_NBK310591.pdf (2010). Accessed 5 Sept 2023. 26269861

[27] World Health Organization. WHO recommendations on maternal and newborn care for a positive postnatal experience. https://iris.who.int/bitstream/handle/10665/352658/9789240045989-eng.pdf (2022). Accessed 5 Sept 2023. 35467813

[28] Ross E, Munoz FM, Edem B, Nan C, Jehan F, Quinn J, et al. Failure to thrive: case definition & guidelines for data collection, analysis, and presentation of maternal immunisation safety data. Vaccine. 2017;35(48 Pt A):6483–91. 29150053 10.1016/j.vaccine.2017.01.051 PMC5714432

[29] Sweet LR, Keech C, Klein NP, Marshall HS, Tagbo BN, Quine D, et al. Respiratory distress in the neonate: case definition & guidelines for data collection, analysis, and presentation of maternal immunization safety data. Vaccine. 2017;35(48 Pt A):6506–17. 29150056 10.1016/j.vaccine.2017.01.046 PMC5710987

[30] Vergnano S, Buttery J, Cailes B, Chandrasekaran R, Chiappini E, Clark E, et al. Neonatal infections: case definition and guidelines for data collection, analysis, and presentation of immunisation safety data. Vaccine. 2016;34(49):6038–46. 27491687 10.1016/j.vaccine.2016.03.046 PMC5139809

[31] Pathirana J, Muñoz FM, Abbing-Karahagopian V, Bhat N, Harris T, Kapoor A, et al. Neonatal death: case definition & guidelines for data collection, analysis, and presentation of immunization safety data. Vaccine. 2016;34(49):6027–37. 27449077 10.1016/j.vaccine.2016.03.040 PMC5139812

[32] DeSilva M, Munoz FM, McMillan M, Kawai AT, Marshall H, Macartney KK, et al. Congenital anomalies: case definition and guidelines for data collection, analysis, and presentation of immunization safety data. Vaccine. 2016;34(49):6015–26. 27435386 10.1016/j.vaccine.2016.03.047 PMC5139892

[33] DeSilva M, Munoz FM, Sell E, Marshall H, Tse Kawai A, Kachikis A, et al. Congenital microcephaly: case definition & guidelines for data collection, analysis, and presentation of safety data after maternal immunisation. Vaccine. 2017;35(48 Pt A):6472–82. 29150052 10.1016/j.vaccine.2017.01.044 PMC5710988

[34] Sell E, Munoz FM, Soe A, Wiznitzer M, Heath PT, Clarke ED, et al. Neonatal encephalopathy: case definition & guidelines for data collection, analysis, and presentation of maternal immunisation safety data. Vaccine. 2017;35(48 Pt A):6501–5. 29150055 10.1016/j.vaccine.2017.01.045 PMC5710979

[35] Cutland CL, Lackritz EM, Mallett-Moore T, Bardají A, Chandrasekaran R, Lahariya C, et al. Low birth weight: case definition & guidelines for data collection, analysis, and presentation of maternal immunization safety data. Vaccine. 2017;35(48 Pt A):6492–500. 29150054 10.1016/j.vaccine.2017.01.049 PMC5710991

[36] Schlaudecker EP, Munoz FM, Bardají A, Boghossian NS, Khalil A, Mousa H, et al. Small for gestational age: case definition & guidelines for data collection, analysis, and presentation of maternal immunisation safety data. Vaccine. 2017;35(48 Pt A):6518–28. 29150057 10.1016/j.vaccine.2017.01.040 PMC5710996

[37] Harrison MS, Eckert LO, Cutland C, Gravett M, Harper DM, McClure EM, et al. Pathways to preterm birth: case definition and guidelines for data collection, analysis, and presentation of immunization safety data. Vaccine. 2016;34(49):6093–101. 27491689 10.1016/j.vaccine.2016.03.054 PMC5139807

[38] Gravett C, Eckert LO, Gravett MG, Dudley DJ, Stringer EM, Mujobu TB, et al. Non-reassuring fetal status: case definition & guidelines for data collection, analysis, and presentation of immunization safety data. Vaccine. 2016;34(49):6084–92. 27461459 10.1016/j.vaccine.2016.03.043 PMC5139811

[39] Kachikis A, Eckert LO, Walker C, Oteng-Ntim E, Guggilla R, Gupta M, et al. Gestational diabetes mellitus: case definition and guidelines for data collection, analysis, and presentation of immunization safety data. Vaccine. 2017;35(48 Pt A):6555–62. 29150061 10.1016/j.vaccine.2017.01.043 PMC5710985

[40] Boatin AA, Eckert LO, Boulvain M, Grotegut C, Fisher BM, King J, et al. Dysfunctional labor: case definition & guidelines for data collection, analysis, and presentation of immunization safety data. Vaccine. 2017;35(48 Pt A):6538–45. 29150059 10.1016/j.vaccine.2017.01.050 PMC5710983

[41] Easter SR, Eckert LO, Boghossian N, Spencer R, Oteng-Ntim E, Ioannou C, et al. Fetal growth restriction: case definition & guidelines for data collection, analysis, and presentation of immunization safety data. Vaccine. 2017;35(48 Pt A):6546–54. 29150060 10.1016/j.vaccine.2017.01.042 PMC5710982

[42] Kerr R, Eckert LO, Winikoff B, Durocher J, Meher S, Fawcus S, et al. Postpartum haemorrhage: case definition and guidelines for data collection, analysis, and presentation of immunization safety data. Vaccine. 2016;34(49):6102–9. 27431424 10.1016/j.vaccine.2016.03.039 PMC5139805

[43] Prabhu M, Eckert LO, Belfort M, Babarinsa I, Ananth CV, Silver RM, et al. Antenatal bleeding: case definition and guidelines for data collection, analysis, and presentation of immunization safety data. Vaccine. 2017;35(48 Pt A):6529–37. 29150058 10.1016/j.vaccine.2017.01.081 PMC5710989

[44] Rouse CE, Eckert LO, Wylie BJ, Lyell DJ, Jeyabalan A, Kochhar S, et al. Hypertensive disorders of pregnancy: case definitions & guidelines for data collection, analysis, and presentation of immunization safety data. Vaccine. 2016;34(49):6069–76. 27426628 10.1016/j.vaccine.2016.03.038 PMC5139806

[45] Patwardhan M, Eckert LO, Spiegel H, Pourmalek F, Cutland C, Kochhar S, et al. Maternal death: case definition and guidelines for data collection, analysis, and presentation of immunization safety data. Vaccine. 2016;34(49):6077–83. 27426627 10.1016/j.vaccine.2016.03.042 PMC5139803

[46] Tavares Da Silva F, Gonik B, McMillan M, Keech C, Dellicour S, Bhange S, et al. Stillbirth: case definition and guidelines for data collection, analysis, and presentation of maternal immunization safety data. Vaccine. 2016;34(49):6057–68. 27431422 10.1016/j.vaccine.2016.03.044 PMC5139804

[47] Munoz FM. Current challenges and achievements in maternal immunization research. Front Immunol. 2018;9: 436. 29559976 10.3389/fimmu.2018.00436 PMC5845678

[48] Bonhoeffer J, Kochhar S, Hirschfeld S, Heath PT, Jones CE, Bauwens J, et al. Global alignment of immunization safety assessment in pregnancy – the GAIA project. Vaccine. 2016;34(49):5993–7. 27751641 10.1016/j.vaccine.2016.07.006

[49] Jones CE, Munoz FM, Spiegel HML, Heininger U, Zuber PLF, Edwards KM, et al. Guideline for collection, analysis and presentation of safety data in clinical trials of vaccines in pregnant women. Vaccine. 2016;34(49):5998–6006. 27481360 10.1016/j.vaccine.2016.07.032 PMC5572188

[50] Stuurman AL, Sharan A, Jahagirdar S, Elango V, Riera-Montes M, Kashyap N, et al. WHO global vaccine safety multi-country collaboration project on safety in pregnancy: assessing the level of diagnostic certainty using standardized case definitions for perinatal and neonatal outcomes and maternal immunization. Vaccine: X. 2021;9: 100123. 34825164 10.1016/j.jvacx.2021.100123 PMC8605263

[51] Jauniaux E, Ayres-de-Campos D, Langhoff-Roos J, Fox KA, Collins S, FIGO placenta accreta diagnosis and management expert consensus panel. Figo classification for the clinical diagnosis of placenta accreta spectrum disorders. Int J Gynaecol Obstet. 2019;146(1):20–4. 31173360 10.1002/ijgo.12761

[52] Jauniaux E, Chantraine F, Silver RM, Langhoff-Roos J, FIGO Placenta Accreta Diagnosis and Management Expert Consensus Panel. Figo consensus guidelines on placenta accreta spectrum disorders: epidemiology. Int J Gynaecol Obstet. 2018;140(3):265–73. 29405321 10.1002/ijgo.12407

[53] Timor-Tritsch IE. Cesarean scar pregnancy. https://www.uptodate.com/contents/cesarean-scar-pregnancy (2024). Accessed 15 Oct 2024.

[54] UNICEF. Levels & trends in child mortality: report 2022. 2023. https://data.unicef.org/resources/levels-and-trends-in-child-mortality/. Accessed 26 Jan 2023.

[55] Walani SR. Global burden of preterm birth. Int J Gynaecol Obstet. 2020;150(1):31–3. 32524596 10.1002/ijgo.13195

[56] World Health Organization. Born too soon: decade of action on preterm birth. 2023. https://www.who.int/publications/i/item/9789240073890. Accessed 21 Nov 2023.

[57] Laelago T, Yohannes T, Tsige G. Determinants of preterm birth among mothers who gave birth in East Africa: systematic review and meta-analysis. Ital J Pediatr. 2020;46(1): 10. 31992346 10.1186/s13052-020-0772-1 PMC6988288

[58] Morokuma S, Michikawa T, Kato K, Sanefuji M, Shibata E, Tsuji M, et al. Non-reassuring foetal status and neonatal irritability in the Japan environment and children’s study: a cohort study. Sci Rep. 2018;8(1): 15853. 30367151 10.1038/s41598-018-34231-y PMC6203769

[59] Noubiap JJ, Bigna JJ, Nyaga UF, Jingi AM, Kaze AD, Nansseu JR, et al. The burden of hypertensive disorders of pregnancy in Africa: a systematic review and meta-analysis. J Clin Hypertens (Greenwich). 2019;21(4):479–88. 30848083 10.1111/jch.13514 PMC8030504

[60] Umesawa M, Kobashi G. Epidemiology of hypertensive disorders in pregnancy: prevalence, risk factors, predictors and prognosis. Hypertens Res. 2017;40(3):213–20. 27682655 10.1038/hr.2016.126

[61] Blencowe H, Krasevec J, De Onis M, Black RE, An X, Stevens GA, et al. National, regional, and worldwide estimates of low birthweight in 2015, with trends from 2000: a systematic analysis. Lancet Glob Health. 2019;7(7):e849–60. 31103470 10.1016/S2214-109X(18)30565-5 PMC6560046

[62] World Health Organization, United Nations Children’s Fund (‎UNICEF). Low birthweight: country, regional and global estimates. https://apps.who.int/iris/handle/10665/43184 (2004). Accessed 6 Jan 2023.

[63] Lee ACC, Katz J, Blencowe H, Cousens S, Kozuki N, Vogel JP, et al. National and regional estimates of term and preterm babies born small for gestational age in 138 low-income and middle-income countries in 2010. Lancet Glob Health. 2013;1(1):e26–36. 25103583 10.1016/S2214-109X(13)70006-8 PMC4221634

[64] Watson G, Dodd C, Munoz FM, Eckert LO, Jones CE, Buttery JP, et al. Applicability of the GAIA maternal and neonatal outcome case definitions for the evaluation of adverse events following vaccination in pregnancy in high-income countries. Pediatr Infect Dis J. 2021;40(12):1127–34. 34596623 10.1097/INF.0000000000003261

[65] United States Agency for International Development. The DHS program| Demographic and health surveys. https://dhsprogram.com. Accessed 17 Oct 2023.

[66] Chmielewska B, Barratt I, Townsend R, Kalafat E, van der Meulen J, Gurol-Urganci I, et al. Effects of the COVID-19 pandemic on maternal and perinatal outcomes: a systematic review and meta-analysis. Lancet Glob Health. 2021;9(6):e759–72. 33811827 10.1016/S2214-109X(21)00079-6 PMC8012052

[67] McClymont E, Albert AY, Alton GD, Boucoiran I, Castillo E, Fell DB, et al. Association of SARS-CoV-2 infection during pregnancy with maternal and perinatal outcomes. JAMA. 2022;327(20):1983–91. 35499852 10.1001/jama.2022.5906 PMC9062768

