# Supplementary material for: A multicountry study to establish rates for pregnancy and neonatal outcomes in low- and middle-income regions
Source: BMC Pregnancy Childbirth. 2026 Jan 14;26:679. doi: 10.1186/s12884-025-08012-1 (PMC13312620; doi:10.1186/s12884-025-08012-1)
Supplement: Supplementary file 1 — Supplementary Material 1. [file 12884_2025_8012_MOESM1_ESM.docx]

# Title page

# A multicountry study to establish rates for pregnancy and neonatal outcomes in low- and middle-income regions

**Running title:** Pregnancy and neonatal outcomes

**SUPPLEMENTARY MATERIAL**

# Supplementary material

# Supplementary Figure 1. Plain language summary


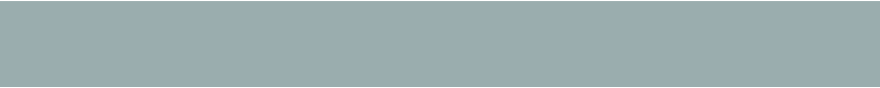


- Vaccines administered to pregnant women can protect both mothers and newborns against severe forms of some diseases.
- To develop safe vaccines for expecting mothers, we need to know how often complications can occur during and after the delivery for both mothers and their newborns.
- In low- and middle-income countries, such complications are not routinely reported and there is insufficient knowledge on the real number of pregnant women and newborns experiencing medical complications.

**What is the context?**


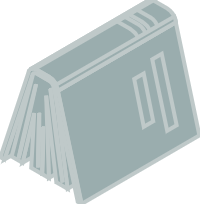


- We conducted a two-year study in 10 countries to determine how often healthy pregnant women and their newborns were diagnosed with a medical complication under normal circumstances.
- Most newborns (2088 out of 2222 pregnancies) were born alive without apparent birth defects. Eighteen newborns were born alive with birth defects.
- Complications most often experienced by pregnant women during labor were births before expected due date (7.5 cases per 100 pregnancies), fetus with abnormal heart rate (6.2 cases per 100 pregnancies) and high blood pressure conditions (5.6 cases per 100 pregnancies).
- Among the newborns, the complications detected most often were low weight (<2500 g) at birth (7.4 cases per 100 births), birth before expected due date (6.7 cases per 100 births), small size for the number of weeks of pregnancy (5.3 cases per 100 births) and birth defects (4.9 cases per 100 births).
- The frequency of the maternal and newborn complications differed among countries.


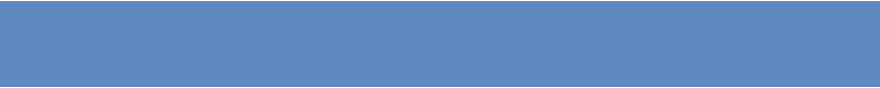


**What is new?**


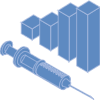


- The results of this study support the interpretation of safety data from clinical trials on candidate vaccines against infectious diseases for pregnant women and newborns from low- and middle-income countries.


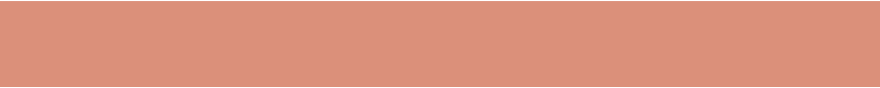


**What is the impact?**


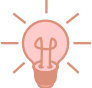


# Supplementary Table 1. Inclusion and exclusion criteria in the study

| **Maternal participants** |
| --- |
| Inclusion criteria   - Healthy pregnant women 18–45 years of age who are ≥24^0/7^ weeks gestational age (GA) at screening and ≤27^6/7^ weeks GA at Visit 1, as established by ultrasound examination and/or last menstrual period date*.   * The level of diagnostic certainty of the GA should be established by using the Global Alignment of Immunization Safety Assessment in Pregnancy GA assessment form.   - Women with pre-pregnancy body mass index ≥18.5 kg/m^2^ and ≤ 39.9 kg/m^2^. - Women whose pregnancy is considered low-risk, based on medical history, obstetric history, and clinical findings during the current pregnancy. - Pregnant women who had no significant findings (such as abnormal fetal morphology, amniotic fluid levels, placenta, or umbilical cord) observed during a Level 2 ultrasound (fetal morphology assessment). - Human immunodeficiency virus (HIV) uninfected pregnant women who have been tested within the past year and have documented HIV negative test results. - Women who give written or witnessed/thumb printed informed consent after the study has been explained according to local regulatory requirements. - The informed consent given at screening should either include consent for both the mother’s participation and participation of the neonate after its birth (if consistent with local regulations/guidelines), or consent for the mother’s participation and expressed willingness to consider permitting the neonate to take part after it has been born (if local regulations/guidelines require parent(s) to provide an additional informed consent after the birth). - Both mother and father should consent if local regulations/guidelines require it. - Women who consent to have cord blood collected at delivery for the purpose of the study. - Women who plan to reside in the study area for at least one year after delivery. - Women who are in good health as determined by the outcome of medical history, physical examination, and clinical judgment of the investigator. - Women who, in the opinion of the investigator can and will comprehend and comply with all study procedures (e.g., return for study follow-up visits, be contactable and available on a regular basis for surveillance).   Exclusion criteria   - Women determined to have one of the following conditions associated with increased risk for a serious obstetrical complication (specify any/all that apply in the electronic case report form [eCRF]): gestational hypertension; gestational diabetes uncontrolled by diet and exercise; pre-eclampsia or eclampsia; multiple pregnancy; intrauterine growth restriction; placenta previa; polyhydramnios; oligohydramnios. - Women determined to have (during the current pregnancy) one of the following infections or conditions associated with risk of adverse outcome (specify any/all that apply in the eCRF): known or suspected syphilis infection, parvovirus B19, rubella infection, primary genital herpes simplex infection, primary cytomegalovirus infection, varicella infection, Zika infection, active tuberculosis infection, incompetent cervix, or cerclage. - Women who have any underlying condition or infection that would predispose them to increased risk for a serious obstetrical complication that is not mentioned above. - Women who have behavioral or cognitive impairment or psychiatric disease that, in the opinion of the investigator, could interfere with the participant’s ability to participate in the study. - Women who have known or suspected impairment of the immune system, an active autoimmune disorder that is not well-controlled, or who are receiving systemic immunosuppressive therapy. - Women participating in any concurrent clinical trial during the current pregnancy. - Women pregnant with a fetus with a confirmed or suspected major congenital anomaly at the time of enrollment. |
| **Neonatal participants** |
| Inclusion criteria:   - Neonates who were *in utero* at the time maternal (and paternal, if required) informed consent was given, and who are liveborn. - If local law requires it: Written or witnessed/thumb printed informed consent for study participation of the neonate obtained from parent(s)/legally authorized representative(s) within 21 days of birth.   Exclusion criterion   - Child in care |

# Supplementary Table 2. Study endpoints

| Pregnancy outcomes | Pregnancy-related events of interest | Neonatal events of interest |
| --- | --- | --- |
| Livebirth with or without congenital anomalies  Fetal death or stillbirth after 22 weeks of gestation with or without congenital anomalies  Antepartum stillbirth  Intrapartum stillbirth  Elective termination with or without congenital anomalies | Maternal death  Hypertensive disorders  Gestational hypertension  Normal and severe pre-eclampsia  Antenatal bleeding  Placenta accreta spectrum disorders  Placental abruption  Cesarean scar pregnancy^a^  Uterine rupture  Postpartum hemorrhage  Fetal growth restriction  Dysfunctional labor  First stage of labor  Second stage of labor  Gestational diabetes mellitus  Non-reassuring fetal status  Pathways to preterm birth  Preterm rupture of membranes  Preterm labor  Provider-initiated preterm birth  Chorioamnionitis  Oligohydramnios  Polyhydramnios  Gestational liver disease  Intrahepatic cholestasis  Acute fatty liver  Maternal sepsis  Other concerning events | Small for gestational age  Low and very low birthweight  Neonatal encephalopathy  Congenital microcephaly  Postnatal  Prenatal  Congenital anomalies  Major external structural defects  Internal structural defects  Functional defects  Neonatal death  In a preterm livebirth (≥28 to <37 weeks gestational age)  In a term livebirth  Neonatal infections  Blood stream infections  Meningitis  Respiratory infection  Respiratory distress in the neonate  Preterm birth  Failure to thrive  Large for gestational age  Macrosomia  Any other concerning neonatal event |

^a^During the conduct of the study, the term “cesarean scar pregnancy” was used to define all hysterotomy scar pregnancies, being considered a source for antenatal bleeding [1]. In 2018, approximatively one year after the protocol was finalized, the International Federation of Gynecology and Obstetrics consensus guidelines recommended the use of the terminology “placenta accreta spectrum disorders” in epidemiologic studies reporting on adherent and invasive placental disorders, which are most likely preceded by a cesarean scar pregnancy [2,3]. Conditions like “cesarean scar pregnancy” and “placenta accreta spectrum disorders” share common histological features and represent a continuum of the same disease [4]. Therefore, the terms “cesarean scar pregnancy” (diagnosed in the first and second trimester of pregnancy) and “placenta accreta spectrum disorders” (diagnosed in the second trimester and beyond) can be considered in future studies to describe different stages of the same disease.

**References**

1. Prabhu M, Eckert LO, Belfort M, Babarinsa I, Ananth CV, Silver RM et al. Antenatal bleeding: case definition and guidelines for data collection, analysis, and presentation of immunization safety data. Vaccine. 2017; 35(48 Pt A):6529-6537.

2. Jauniaux E, Ayres-de-Campos D, Langhoff-Roos J, Fox KA, Collins S, FIGO Placenta Accreta Diagnosis and Management Expert Consensus Panel. FIGO classification for the clinical diagnosis of placenta accreta spectrum disorders. Int J Gynaecol Obstet. 2019; 146(1):20-24

3. Jauniaux E, Chantraine F, Silver RM, Langhoff-Roos J, FIGO Placenta Accreta Diagnosis and Management Expert Consensus Panel. FIGO consensus guidelines on placenta accreta spectrum disorders: Epidemiology. Int J Gynaecol Obstet. 2018; 140(3):265-273.

4. Timor-Tritsch IE: Cesarean scar pregnancy. https://www.uptodate.com/contents/cesarean-scar-pregnancy (2024). Accessed 15 October 2024.

# Supplementary Table 3. Standard definitions for maternal and neonatal EOIs not defined as events in GAIA case definition

| EOIs | Definition |
| --- | --- |
| Maternal EOIs | |
| Chorioamnionitis | Chorioamnionitis also known as intra-amniotic infection is an inflammation of the fetal membranes due to a bacterial infection. Clinical signs and symptoms of chorioamnionitis include the following:  • Fever (an intrapartum temperature >37.8 ºC)  • Significant maternal tachycardia (>120 bpm)  • Fetal tachycardia (>160–180 bpm)  • Purulent or foul-smelling amniotic fluid or vaginal discharge  • Uterine tenderness  • Maternal leukocytosis (total blood leukocyte count >15000–18000 cells/μL)  Of these criteria, intrapartum maternal fever appears to be the most frequent. When at least two of the forementioned criteria are present, the risk of neonatal sepsis is increased. Each clinical sign and symptom of chorioamnionitis, however, is by itself of low predictive value. |
| Oligohydramnios | AFI <8 cm or DVP <2 cm in the presence of intact membranes without concern for fetal anomalies contributing to its etiology. |
| Polyhydramnios | Polyhydramnios is the presence of excess amniotic fluid in the uterus. By definition, polyhydramnios is diagnosed if the deepest vertical pool is >8 cm or AFI is more than 95^th^ percentile for the corresponding gestational age. |
| Gestational liver disease (ICP) [1] | Intrahepatic cholestasis (obstetric cholestasis) should be suspected when pruritis develops during pregnancy in the absence of a rash. Lab evidence of cholestasis includes elevated bile acids (Glyco and Taurochenodeoxycholic Acid) (>10 µmol/L). Up to 60% of patients will have elevated transaminases and 20% of patients will have increased direct bilirubin levels.  Jaundice may or may not be present. ICP typically is transient and resolves after delivery. Women that had ICP in previous pregnancy have higher risk to developing ICP in the following pregnancies or other hepatobiliary disorders in later life. ICP is associated with adverse fetal outcome like meconium-stained liquor, fetal asphyxia, spontaneous preterm delivery, and intrauterine death. |
| Gestational liver disease (AFLP) [2] | AFLP is a rare, potentially fatal complication that occurs in the third trimester or early postpartum period. AFLP is characterized by microvesicular fatty infiltration of hepatocytes without any inflammation or necrosis. Most frequent signs and symptoms are the following:  • Jaundice  • Abdominal pain (usually right upper quadrat, midepigastric or radiating to back)  • Central nervous system (altered sensorium, confusion, disorientation, psychosis, restlessness, seizures or even coma)  • Disseminated intravascular coagulation  • Nausea and vomiting  • Gastrointestinal bleeding  • Acute renal failure  • Oliguria  • Tachycardia  • Late onset pyrexia  • Hypoglycemia  • ALT<500 units/L  • Hyperbilirubinemia, elevated ammonia, leukocytosis, hypofibrinogenemia  Ultrasound examination and computed tomography may demonstrate fatty infiltration of the liver but are not sufficient for diagnosis. |
| Maternal sepsis [3] | Maternal sepsis is a life-threatening condition defined as organ dysfunction resulting from infection during pregnancy, childbirth, post-abortion, or postpartum period.  Organ dysfunction can be identified as an acute change in total SOFA score ≥2 points consequent to the infection.  The baseline SOFA score can be assumed to be zero in patients not known to have pre-existing organ dysfunction.  A SOFA score ≥2 reflects an overall mortality risk of approximately 10% in a general hospital population with suspected infection. Even patients presenting with modest dysfunction can deteriorate further, emphasizing the seriousness of this condition and the need for prompt and appropriate intervention, if not already being instituted. |
| Neonatal EOIs | |
| Large for gestational age | BW >90% for neonates of same gestational age in same population (>4000 g at term). |
| Macrosomia | BW >4000 g. |

EOI, events of interest; GAIA, Global Alignment of Immunization Safety Assessment in Pregnancy; bpm, beats per minute; AFI, amniotic fluid index; DVP, deepest vertical pocket; ICP, intrahepatic cholestasis of pregnancy; AFLP, acute fatty liver of pregnancy; ALT, alanine transaminase; SOFA, sequential (sepsis-related) organ failure assessment; BW, birthweight.

**References:**

1. Geenes V, Williamson C. Intrahepatic cholestasis of pregnancy. *World J Gastroenterol* 2009; 15:2049-66.

2. Ko H, Yoshida EM. Acute fatty liver of pregnancy. *Can J Gastroenterol* 2006; 20:25-30.

3. Bonet M, Nogueira Pileggi V, Rijken MJ, Coomarasamy A, Lissauer D, Souza JP, et al. Towards a consensus definition of maternal sepsis: results of a systematic review and expert consultation. *Reprod Health* 2017; 14:67.

# Supplementary Table 4. Baseline characteristics for maternal and neonatal participants (maternal and neonatal enrolled set)

| Maternal set | Bangladesh (N=207) | Malaysia (N=170) | Philippines (N=279) | Thailand (N=262) | South Africa (N=399) | Argentina (N=324) | Brazil (N=198) | Colombia (N=281) | Mexico (N=125) | Panama (N=66) | Overall (N=2311) |
| --- | --- | --- | --- | --- | --- | --- | --- | --- | --- | --- | --- |
| Age category, n (%) | | | | | | | | | | | |
| 18–34 years | 200 (96.6) | 130 (76.5) | 237 (84.9) | 197 (75.2) | 372 (93.2) | 263 (81.2) | 175 (88.4) | 246 (87.5) | 113 (90.4) | 61 (92.4) | 1994 (86.3) |
| 35–39 years | 5 (2.4) | 30 (17.6) | 32 (11.5) | 55 (21.0) | 27 (6.8) | 52 (16.0) | 21 (10.6) | 27 (9.6) | 9 (7.2) | 4 (6.1) | 262 (11.3) |
| ≥40 years | 2 (1.0) | 10 (5.9) | 10 (3.6) | 10 (3.8) | 0 (0.0) | 9 (2.8) | 2 (1.0) | 8 (2.8) | 3 (2.4) | 1 (1.5) | 55 (2.4) |
| Ethnicity, n (%) | | | | | | | | | | | |
| African/African American | 0 (0.0) | 0 (0.0) | 0 (0.0) | 0 (0.0) | 281 (70.4) | 0 (0.0) | 34 (17.2) | 59 (21.0) | 0 (0.0) | 4 (6.1) | 378 (16.4) |
| American Indian/Alaska Native | 0 (0.0) | 0 (0.0) | 0 (0.0) | 0 (0.0) | 1 (0.3) | 0 (0.0) | 0 (0.0) | 4 (1.4) | 44 (35.2) | 2 (3.0) | 51 (2.2) |
| Asian | 207 (100) | 170 (100) | 279 (100) | 262 (100) | 0 (0.0) | 0 (0.0) | 1 (0.5) | 0 (0.0) | 0 (0.0) | 0 (0.0) | 919 (39.8) |
| White^a^ | 0 (0.0) | 0 (0.0) | 0 (0.0) | 0 (0.0) | 0 (0.0) | 324 (100) | 83 (41.9) | 0 (0.0) | 1 (0.8) | 0 (0.0) | 408 (17.7) |
| Other | 0 (0.0) | 0 (0.0) | 0 (0.0) | 0 (0.0) | 117 (29.3) | 0 (0.0) | 80 (40.4) | 218 (77.6) | 80 (64.0) | 60 (90.9) | 555 (24.0) |
| Highest education level^b^, n (%) | | | | | | | | | | | |
| Less than primary | 26 (12.6) | 0 (0.0) | 1 (0.4) | 1 (0.4) | 1 (0.3) | 2 (0.6) | 10 (5.1) | 0 (0.0) | 0 (0.0) | 1 (1.5) | 42 (1.8) |
| Primary | 25 (12.1) | 5 (2.9) | 13 (4.7) | 1 (0.4) | 5 (1.3) | 15 (4.6) | 8 (4.0) | 21 (7.5) | 3 (2.4) | 1 (1.5) | 97 (4.2) |
| Secondary | 122 (58.9) | 94 (55.3) | 169 (60.6) | 76 (29.0) | 311 (77.9) | 215 (66.4) | 151 (76.2) | 181 (64.4) | 48 (38.4) | 48 (72.7) | 1415 (61.2) |
| Short cycle tertiary | 0 (0.0) | 38 (22.4) | 12 (4.3) | 15 (5.7) | 60 (15.0) | 22 (6.8) | 10 (5.1) | 19 (6.8) | 22 (17.6) | 0 (0.0) | 198 (8.6) |
| Bachelor’s | 8 (3.9) | 27 (15.9) | 82 (29.4) | 139 (53.1) | 15 (3.8) | 67 (20.7) | 18 (9.1) | 44 (15.7) | 50 (40.0) | 15 (22.7) | 465 (20.1) |
| Master’s | 1 (0.5) | 5 (2.9) | 1 (0.4) | 29 (11.1) | 0 (0.0) | 0 (0.0) | 0 (0.0) | 11 (3.9) | 2 (1.6) | 1 (1.5) | 50 (2.2) |
| Doctorate or equivalent | 0 (0.0) | 0 (0.0) | 0 (0.0) | 0 (0.0) | 0 (0.0) | 1 (0.3) | 0 (0.0) | 0 (0.0) | 0 (0.0) | 0 (0.0) | 1 (0.04) |
| Not elsewhere classified | 25 (12.1) | 1 (0.6) | 1 (0.4) | 1 (0.4) | 7 (1.8) | 2 (0.6) | 1 (0.5) | 5 (1.8) | 0 (0.0) | 0 (0.0) | 43 (1.9) |
| Household environment, n (%) | | | | | | | | | | | |
| Urban | 105 (50.7) | 151 (88.8) | 277 (99.3) | 223 (85.1) | 62 (15.5) | 319 (98.5) | 197 (99.5) | 274 (97.5) | 120 (96.0) | 61 (92.4) | 1789 (77.4) |
| Suburban | 25 (12.1) | 19 (11.2) | 1 (0.4) | 36 (13.7) | 299 (74.9) | 3 (0.9) | 1 (0.5) | 0 (0.0) | 4 (3.2) | 2 (3.0) | 390 (16.9) |
| Rural | 77 (37.2) | 0 (0.0) | 1 (0.4) | 3 (1.1) | 38 (9.5) | 2 (0.6) | 0 (0.0) | 7 (2.5) | 1 (0.8) | 3 (4.5) | 132 (5.7) |
| Smoking status during current pregnancy, n (%) | | | | | | | | | | | |
| Yes, in trimester 1 | 0 (0.0) | 0 (0.0) | 11 (3.9) | 3 (1.1) | 68 (17.0) | 18 (5.6) | 17 (8.6) | 11 (3.9) | 3 (2.4) | 0 (0.0) | 131 (5.7) |
| Yes, in trimester 2 | 0 (0.0) | 0 (0.0) | 5 (1.8) | 0 (0.0) | 57 (14.3) | 3 (0.9) | 8 (4.0) | 2 (0.7) | 1 (0.8) | 0 (0.0) | 76 (3.3) |
| No | 207 (100) | 170 (100) | 267 (95.7) | 259 (98.9) | 329 (82.5) | 305 (94.1) | 181 (91.4) | 270 (96.1) | 122 (97.6) | 66 (100) | 2176 (94.2) |
| Alcohol consumption during current pregnancy, n (%) | | | | | | | | | | | |
| Yes, in trimester 1 | 0 (0.0) | 0 (0.0) | 13 (4.7) | 4 (1.5) | 51 (12.8) | 17 (5.2) | 42 (21.2) | 13 (4.6) | 4 (3.2) | 1 (1.5) | 145 (6.3) |
| Yes, in trimester 2 | 0 (0.0) | 0 (0.0) | 2 (0.7) | 1 (0.4) | 26 (6.5) | 3 (0.9) | 11 (5.6) | 0 (0.0) | 0 (0.0) | 0 (0.0) | 43 (1.9) |
| No | 207 (100) | 170 (100) | 265 (95.0) | 257 (98.1) | 339 (85.0) | 305 (94.1) | 151 (76.3) | 268 (95.4) | 121 (96.8) | 65 (98.5) | 2148 (92.9) |
| Living in country/region with Zika transmission, n (%) | | | | | | | | | | | |
| Yes | 206 (99.5) | 163 (95.9) | 275 (98.6) | 258 (98.5) | 0 (0.0) | 307 (94.8) | 197 (99.5) | 272 (96.8) | 120 (96.0) | 65 (98.5) | 1863 (80.6) |
| No | 1 (0.5) | 7 (4.1) | 4 (1.4) | 4 (1.5) | 399 (100) | 17 (5.2) | 1 (0.5) | 9 (3.2) | 5 (4.0) | 1 (1.5) | 448 (19.4) |
| Neonatal set | Bangladesh (N=195) | Malaysia (N=167) | Philippines (N=259) | Thailand (N=258) | South Africa (N=385) | Argentina (N=306) | Brazil (N=185) | Colombia (N=268) | Mexico (N=97) | Panama (N=61) | Overall (N=2181) |
| Gestational age at birth | | | | | | | | | | | |
| Mean±SD (weeks) | 38.2±1.6 | 38.1±1.9 | 38.4±1.5 | 38.4±1.2 | 38.8±2.0 | 38.5±1.6 | 38.9±1.6 | 38.6±1.6 | 38.3±1.8 | 38.9±1.5 | 38.5±1.7 |
| Missing data, n | 0 | 0 | 7 | 0 | 1 | 1 | 0 | 1 | 0 | 1 | 11 |
| Male sex, n (%) | 108 (55.4) | 91 (54.5) | 146 (56.4) | 135 (52.3) | 203 (52.7) | 136 (44.4) | 96 (51.9) | 132 (49.3) | 52 (53.6) | 33 (54.1) | 1132 (51.9) |
| Length | | | | | | | | | | | |
| Mean±SD (cm) | 47.5±2.3 | 48.6±2.6 | 48.9±2.7 | 49.6±2.0 | 49.8±3.4 | 48.9±2.5 | 49.1±2.1 | 49.8±2.4 | 49.6±3.0 | 50±2.6 | 49.2±2.7 |
| Missing data. n | 15 | 0 | 14 | 0 | 26 | 1 | 0 | 2 | 0 | 1 | 59 |
| Birthweight | | | | | | | | | | | |
| Mean±SD (kg) | 2.8±0.4 | 3.0±0.5 | 2.9±0.4 | 3.1±0.4 | 3.1±0.5 | 3.3±0.5 | 3.3±0.5 | 3.2±0.4 | 3.1±0.5 | 3.3±0.4 | 3.1±0.5 |
| Missing data, n | 12 | 0 | 5 | 0 | 9 | 1 | 0 | 1 | 0 | 1 | 29 |
| Apgar score at 5 min | | | | | | | | | | | |
| Mean±SD | 9.2±0.9 | 9.4±0.8 | 9.0±0.2 | 9.7±0.5 | 9.6±0.7 | 9.3±0.8 | 9.4±0.8 | 9.4±1.2 | 9.1±0.5 | 9.0±0.1 | 9.4±0.8 |
| Missing data, n | 20 | 47 | 23 | 0 | 40 | 3 | 0 | 38 | 0 | 1 | 172 |
| Breast feeding, n (%) | | | | | | | | | | | |
| Yes | 191 (97.9) | 162 (97.0) | 248 (95.8) | 252 (97.7) | 372 (96.6) | 303 (99.0) | 183 (98.9) | 267 (99.6) | 95 (97.9) | 58 (95.1) | 2131 (97.7) |
| No | 1 (0.5) | 1 (0.6) | 2 (0.8) | 1 (0.4) | 3 (0.8) | 1 (0.3) | 1 (0.5) | 0 (0.0) | 1 (1.0) | 1 (1.6) | 12 (0.6) |
| Missing | 3 (1.5) | 4 (2.4) | 9 (3.5) | 5 (1.9) | 10 (2.6) | 2 (0.7) | 1 (0.5) | 1 (0.4) | 1 (1.0) | 2 (3.3) | 38 (1.7) |

N, number of participants; n (%), number and percentage of participants in a given category; SD, standard deviation.

Notes:

^a^White-Arabic/North African ethnicity and White-Caucasian/European ethnicity

^b^Bachelor’s degree or higher.

# Supplementary Table 5. Percentage of pregnancy outcomes from first visit to 42 days after delivery, overall and by country (maternal analysis set)

|  | % (95 confidence interval) | | | | | | | | | | |
| --- | --- | --- | --- | --- | --- | --- | --- | --- | --- | --- | --- |
| Outcome | Bangladesh (N=201) | Malaysia  (N=162) | Philippines (N=258) | Thailand  (N=260) | South Africa  (N=388) | Argentina  (N=301) | Brazil  (N=196) | Colombia  (N=269) | Mexico  (N=121) | Panama  (N=66) | Overall  (N=2222^a^) |
| Livebirth with no congenital anomalies | 91.5  (86.8, 95.0) | 97.5  (93.8, 99.3) | 92.6  (88.7, 95.5) | 96.5  (93.5, 98.4) | 93.6  (90.6, 95.8) | 91.0  (87.2, 94.0) | 95.4  (91.5, 97.9) | 95.9  (92.8, 97.9) | 92.6  (86.3, 96.5) | 93.9  (85.2, 98.3) | 94.0  (92.9, 94.9) |
| Livebirth with congenital anomalies | 0.5  (0.0, 2.7) | 1.2  (0.1, 4.4) | 0.4  (0.0, 2.1) | 1.9  (0.6, 4.4) | 0.8  (0.2, 2.2) | 1.3  (0.4, 3.4) | 0.5  (0.0, 2.8) | 0.0  (0.0, 1.4) | 0.8  (0.0, 4.5) | 0.0  (0.0, 5.4) | 0.8  (0.5, 1.3) |
| Antepartum fetal death/stillbirth with no congenital anomalies | 0.0  (0.0, 1.8) | 0.6  (0.0, 3.4) | 0.0  (0.0, 1.4) | 0.0  (0.0, 1.4) | 0.8  (0.2, 2.2) | 0.7  (0.1, 2.4) | 1.0  (0.1, 3.6) | 0.0  (0.0, 1.4) | 0.0  (0.0, 3.0) | 0.0  (0.0, 5.4) | 0.4  (0.2, 0.7) |
| Intrapartum fetal death/stillbirth with no congenital anomalies | 0.0  (0.0, 1.8) | 0.0  (0.0, 2.3) | 0.8  (0.1, 2.8) | 0.0  (0.0, 1.4) | 0.5  (0.1, 1.8) | 0.3  (0.0, 1.8) | 0.0  (0.0, 1.9) | 0.0  (0.0, 1.4) | 0.0  (0.0, 3.0) | 0.0  (0.0, 5.4) | 0.2  (0.1, 0.5) |
| Antepartum fetal death/stillbirth with congenital anomalies | 0.0  (0.0, 1.8) | 0.0  (0.0, 2.3) | 0.0  (0.0, 1.4) | 0.0  (0.0, 1.4) | 0.0  (0.0, 0.9) | 0.0  (0.0, 1.2) | 0.0  (0.0, 1.9) | 0.0  (0.0, 1.4) | 0.0  (0.0, 3.0) | 0.0  (0.0, 5.4) | 0.0  (0.0, 0.2) |
| Intrapartum fetal death/stillbirth with congenital anomalies | 0.0  (0.0, 1.8) | 0.0  (0.0, 2.3) | 0.4  (0.0, 2.1) | 0.0  (0.0, 1.4) | 0.0  (0.0, 0.9) | 0.0  (0.0, 1.2) | 0.0  (0.0, 1.9) | 0.0  (0.0, 1.4) | 0.0  (0.0, 3.0) | 0.0  (0.0, 5.4) | 0.05  (0.0, 0.3) |
| Elective/therapeutic termination with no congenital anomalies | 1.5  (0.3, 4.3) | 0.0  (0.0, 2.3) | 0.0  (0.0, 1.4) | 0.4  (0.0, 2.1) | 0.0  (0.0, 0.9) | 2.0  (0.7, 4.3) | 0.0  (0.0, 1.9) | 0.0  (0.0, 1.4) | 0.0  (0.0, 3.0) | 1.5  (0.0, 8.2) | 0.5  (0.2, 0.9) |
| Elective/therapeutic termination with congenital anomalies | 0.0  (0.0, 1.8) | 0.0  (0.0, 2.3) | 0.0  (0.0, 1.4) | 0.0  (0.0, 1.4) | 0.0  (0.0, 0.9) | 0.0  (0.0, 1.2) | 0.0  (0.0, 1.9) | 0.0  (0.0, 1.4) | 0.8  (0.0, 4.5) | 0.0  (0.0, 5.4) | 0.05  (0.0, 0.3) |
| Missing | 0.0  (0.0, 1.8) | 0.0  (0.0, 2.3) | 1.2  (0.2, 3.4) | 0.0  (0.0, 1.4) | 0.5  (0.1, 1.8) | 0.0  (0.0, 1.2) | 0.0  (0.0, 1.9) | 0.0  (0.0, 1.4) | 0.0  (0.0, 3.0) | 0.0  (0.0, 5.4) | 0.2  (0.1, 0.5) |

%, percentage of participants with the pregnancy outcome; N, number of participants included in the analyses.

Note: ^a^Eighty-five (3.8%) participants in the analysis set did not attend the delivery visit and therefore no outcome was reported for their pregnancies.

# Supplementary Table 6. Percentage of maternal EOIs occurring from first visit to 42 days post-delivery, according to GAIA levels of diagnostic certainty, overall and by country (maternal analysis set)

|  |  | % (95% confidence interval) | | | | | | | | | | |
| --- | --- | --- | --- | --- | --- | --- | --- | --- | --- | --- | --- | --- |
| EOI | GAIA level | Bangladesh  (N=201) | Malaysia  (N=162) | Philippines  (N=258) | Thailand  (N=260) | South Africa  (N=388) | Argentina  (N=301) | Brazil  (N= 196) | Colombia  (N= 269) | Mexico  (N=121) | Panama  (N=66) | Overall  (N=2222) |
| Maternal death | 3 | 0.5  (0.0, 2.7) | 0.0  (0.0, 2.3) | 0.0  (0.0, 1.4) | 0.0  (0.0, 1.4) | 0.0  (0.0, 1.0) | 0.0  (0.0, 1.2) | 0.0  (0.0, 1.9) | 0.0  (0.0, 1.4) | 0.0  (0.0, 3.0) | 0.0  (0.0, 5.4) | 0.1  (0.0, 0.3) |
| Hypertensive disorders of pregnancy | Total | 0.5  (0.0, 2.7) | 2.5  (0.7, 6.2) | 4.7  (2.4, 8.0) | 2.7  (1.1, 5.5) | 9.8  (7.0, 13.2) | -  (4.6, 10.9) | 9.2  (5.5, 14.1) | 6.3  (3.7, 9.9) | 3.3  (0.9, 8.3) | 3.0  (0.4, 10.5) | 5.6  (4.7, 6.7) |
| Gestational hypertension | 1 | 0.0  (0.0, 1.8) | 0.6  (0.0, 3.4) | 0.4  (0.0, 2.1) | 0.8  (0.1, 2.8) | 1.0  (0.3, 2.6) | -  (0.5, 3.8) | 3.6  (1.5, 7.2) | 2.2  (0.8, 4.8) | 0.8  (0.0, 4.5) | 0.0  (0.0, 5.4) | 1.2  (0.8, 1.8) |
|  | 2 | 0.0  (0.0, 1.8) | 1.2  (0.2, 4.4) | 0.8  (0.1, 2.8) | 0.4  (0.0, 2.1) | 1.8  (0.7, 3.7) | -  (1.2, 5.2) | 0.5  (0.0, 2.8) | 0.0  (0.0, 1.4) | 1.7  (0.2, 5.8) | 0.0  (0.0, 5.4) | 1.0  (0.7, 1.6) |
|  | Insufficient evidence | 0.5  (0.0, 2.7) | 0.0  (0.0, 2.3) | 0.8  (0.1, 2.8) | 0.0  (0.0, 1.4) | 3.1  (1.6, 5.3) | -  (0.1, 2.4) | 1.5  (0.3, 4.4) | 0.4  (0.0, 2.1) | 0.0  (0.0, 3.0) | 0.0  (0.0, 5.4) | 1.0  (0.6, 1.4) |
|  | Unavailable | 0.0  (0.0, 1.8) | 0.0  (0.0, 2.3) | 0.4  (0.0, 2.1) | 0.0  (0.0, 1.4) | 0.0  (0.0, 1.0) | 0.0  (0.0, 1.2) | 0.0  (0.0, 1.9) | 0.0  (0.0, 1.4) | 0.0  (0.0, 3.0) | 0.0  (0.0, 5.4) | 0.1  (0.0, 0.3) |
| Pre-eclampsia | 1 | 0.0  (0.0, 1.8) | 0.6  (0.0, 3.4) | 0.4  (0.0, 2.1) | 0.4  (0.0, 2.1) | 0.3  (0.0, 1.4) | -  (0.2, 2.9) | 1.5  (0.3, 4.4) | 2.6  (1.1, 5.3) | 0.0  (0.0, 3.0) | 0.0  (0.0, 5.4) | 0.8  (0.5, 1.2) |
|  | 2 | 0.0  (0.0, 1.8) | 0.0  (0.0, 2.3) | 0.8  (0.1, 2.8) | 0.4  (0.0, 2.1) | 1.8  (0.7, 3.7) | 0.0  (0.0, 1.2) | 0.5  (0.0, 2.8) | 0.0  (0.0, 1.4) | 0.0  (0.0, 3.0) | 1.5  (0.0, 8.2) | 0.5  (0.3, 0.9) |
|  | Insufficient evidence | 0.0  (0.0, 1.8) | 0.0  (0.0, 2.3) | 0.4  (0.0, 2.1) | 0.0  (0.0, 1.4) | 1.0  (0.3, 2.6) | 0.0  (0.0, 1.2) | 1.0  (0.1, 3.6) | 0.4  (0.0, 2.1) | 0.0  (0.0, 3.0) | 0.0  (0.0, 5.4) | 0.4  (0.2, 0.7) |
| Pre-eclampsia with severe features | 1 | 0.0  (0.0, 1.8) | 0.0  (0.0, 2.3) | 0.0  (0.0, 1.4) | 0.8  (0.1, 2.8) | 0.5  (0.1, 1.9) | -  (0.4, 3.4) | 0.5  (0.0, 2.8) | 0.7  (0.1, 2.7) | 0.8  (0.0, 4.5) | 1.5  (0.0, 8.2) | 0.6  (0.3, 1.0) |
|  | Insufficient evidence | 0.0  (0.0, 1.8) | 0.0  (0.0, 2.3) | 0.8  (0.1, 2.8) | 0.0  (0.0, 1.4) | 0.3  (0.0, 1.4) | 0.0  (0.0, 1.2) | 0.0  (0.0, 1.9) | 0.0  (0.0, 1.4) | 0.0  (0.0, 3.0) | 0.0  (0.0, 5.4) | 0.1  (0.0, 0.4) |
| Antenatal bleeding | Total | 0.0  (0.0, 1.8) | 0.6  (0.0, 3.4) | 0.0  (0.0, 1.4) | 0.0  (0.0, 1.4) | 1.3  (0.4, 3.0) | -  (0.2, 2.9) | 0.0  (0.0, 1.9) | 0.7  (0.1, 2.7) | 0.0  (0.0, 3.0) | 0.0  (0.0, 5.4) | 0.5  (0.3, 0.9) |
| Placenta accreta spectrum disorders | 2 | 0.0  (0.0, 1.8) | 0.0  (0.0, 2.3) | 0.0  (0.0, 1.4) | 0.0  (0.0, 1.4) | 0.0  (0.0, 1.0) | 0.0  (0.0, 1.2) | 0.0  (0.0, 1.9) | 0.4  (0.0, 2.1) | 0.0  (0.0, 3.0) | 0.0  (0.0, 5.4) | 0.1  (0.0, 0.3) |
| Placental abruption | 1 | 0.0  (0.0, 1.8) | 0.0  (0.0, 2.3) | 0.0  (0.0, 1.4) | 0.0  (0.0, 1.4) | 0.0  (0.0, 1.0) | -  (0.2, 2.9) | 0.0  (0.0, 1.9) | 0.0  (0.0, 1.4) | 0.0  (0.0, 3.0) | 0.0  (0.0, 5.4) | 0.1  (0.0, 0.4) |
|  | 2 | 0.0  (0.0, 1.8) | 0.6  (0.0, 3.4) | 0.0  (0.0, 1.4) | 0.0  (0.0, 1.4) | 0.5  (0.1, 1.9) | 0.0  (0.0, 1.2) | 0.0  (0.0, 1.9) | 0.4  (0.0, 2.1) | 0.0  (0.0, 3.0) | 0.0  (0.0, 5.4) | 0.2  (0.1, 0.5) |
|  | Incorrect classification | 0.0  (0.0, 1.8) | 0.0  (0.0, 2.3) | 0.0  (0.0, 1.4) | 0.0  (0.0, 1.4) | 0.8  (0.2, 2.2) | 0.0  (0.0, 1.2) | 0.0  (0.0, 1.9) | 0.0  (0.0, 1.4) | 0.0  (0.0, 3.0) | 0.0  (0.0, 5. 4) | 0.1  (0.0, 0.4) |
| Postpartum hemorrhage | 1 | 0.5  (0.0, 2.7) | 0.0  (0.0, 2.3) | 0.0  (0.0, 1.4) | 0.0  (0.0, 1.4) | 0.0  (0.0, 1.0) | 0.0  (0.0, 1.2) | 1.5  (0.3, 4.4) | 0.4  (0.0, 2.1) | 0.0  (0.0, 3.0) | 0.0  (0.0, 5.4) | 0.2  (0.1, 0.5) |
|  | 2 | 0.0  (0.0, 1.8) | 1.9  (0.4, 5.3) | 0.4  (0.0, 2.1) | 0.4  (0.0, 2.1) | 2.3  (1.1, 4.4) | -  (0.0, 1.8) | 0.0  (0.0, 1.9) | 0.0  (0.0, 1.4) | 1.7  (0.2, 5.8) | 0.0  (0.0, 5.4) | 0.8  (0.5, 1.2) |
|  | 3 | 0.0  (0.0, 1.8) | 0.6  (0.0, 3.4) | 0.0  (0.0, 1.4) | 0.4  (0.0, 2.1) | 0.3  (0.0, 1.4) | -  (0.0, 1.8) | 0.0  (0.0, 1.9) | 0.4  (0.0, 2.1) | 0.8  (0.0, 4.5) | 0.0  (0.0, 5.4) | 0.3  (0.1, 0.6) |
|  | Incorrect classification | 0.0  (0.0, 1.8) | 0.0  (0.0, 2.3) | 0.0  (0.0, 1.4) | 0.0  (0.0, 1.4) | 0.8  (0.2, 2.2) | 0.0  (0.0, 1.2) | 0.5  (0.0, 2.8) | 0.4  (0.0, 2.1) | 0.0  (0.0, 3.0) | 0.0  (0.0, 5.4) | 0.2  (0.1, 0.5) |
|  | Unavailable | 0.0  (0.0, 1.8) | 2.5  (0.7, 6.2) | 0.0  (0.0, 1.4) | 0.0  (0.0, 1.4) | 0.0  (0.0, 1.0) | 0.0  (0.0, 1.2) | 0.0  (0.0, 1.9) | 0.0  (0.0, 1.4) | 0.0  (0.0, 3.0) | 0.0  (0.0, 5.4) | 0.2  (0.1, 0.5) |
| Fetal growth restriction | 1a | 0.0  (0.0, 1.8) | 0.6  (0.0, 3.4) | 0.0  (0.0, 1.4) | 0.0  (0.0, 1.4) | 0.0  (0.0, 1.0) | -  (0.1, 2.4) | 0.5  (0.0, 2.8) | 0.4  (0.0, 2.1) | 0.0  (0.0, 3.0) | 0.0  (0.0, 5.4) | 0.2  (0.1, 0.5) |
|  | 1b | 0.0  (0.0, 1.8) | 0.0  (0.0, 2.3) | 0.0  (0.0, 1.4) | 0.4  (0.0, 2.1) | 0.0  (0.0, 1.0) | 0.0  (0.0, 1.2) | 0.0  (0.0, 1.9) | 0.4  (0.0, 2.1) | 0.0  (0.0, 3.0) | 0.0  (0.0, 5.4) | 0.1  (0.0, 0.3) |
|  | 2a | 0.5  (0.0, 2.7) | 0.0  (0.0, 2.3) | 0.0  (0.0, 1.4) | 0.0  (0.0, 1.4) | 0.0  (0.0, 1.0) | 0.0  (0.0, 1.2) | 0.5  (0.0, 2.8) | 0.0  (0.0, 1.4) | 0.0  (0.0, 3.0) | 0.0  (0.0, 5.4) | 0.1  (0.0, 0.3) |
|  | 2b | 0.0  (0.0, 1.8) | 0.0  (0.0, 2.3) | 0.0  (0.0, 1.4) | 0.4  (0.0, 2.1) | 0.0  (0.0, 1.0) | 0.0  (0.0, 1.2) | 1.0  (0.1, 3.6) | 1.1  (0.2, 3.2) | 0.8  (0.0, 4.5) | 0.0  (0.0, 5.4) | 0.3  (0.1, 0.7) |
|  | Insufficient evidence | 0.0  (0.0, 1.8) | 0.0  (0.0, 2.3) | 0.0  (0.0, 1.4) | 0.0  (0.0, 1.4) | 0.5  (0.1, 1.9) | 0.0  (0.0, 1.2) | 0.5  (0.0, 2.8) | 0.0  (0.0, 1.4) | 0.0  (0.0, 3.0) | 0.0  (0.0, 5.4) | 0.1  (0.0, 0.4) |
| Dysfunctional labor-1^st^ stage of labor | 1 | 1.5  (0.3, 4.3) | 0.0  (0.0, 2.3) | 1.6  (0.4, 3.9) | 2.3  (0.9, 5.0) | 0.5  (0.1, 1.9) | -  (0.4, 3.4) | 3.6  (1.5, 7.2) | 3.0  (1.3, 5.8) | 0.0  (0.0, 3.0) | 0.0  (0.0, 5.4) | 1.5  (1.1, 2.1) |
|  | 2 | 0.0  (0.0, 1.8) | 0.0  (0.0, 2.3) | 0.8  (0.1, 2.8) | 0.8  (0.1, 2.8) | 0.8  (0.2, 2.2) | 0.0  (0.0, 1.2) | 0.0  (0.0, 1.9) | 0.4  (0.0, 2.1) | 0.0  (0.0, 3.0) | 1.5  (0.0, 8.2) | 0.4  (0.2, 0.8) |
|  | Incorrect classification | 0.0  (0.0, 1.8) | 0.0  (0.0, 2.3) | 0.0  (0.0, 1.4) | 0.0  (0.0, 1.4) | 1.0  (0.3, 2.6) | 0.0  (0.0, 1.2) | 0.0  (0.0, 1.9) | 0.7  (0.1, 2.7) | 0.0  (0.0, 3.0) | 0.0  (0.0, 5.4) | 0.3  (0.1, 0.6) |
| Dysfunctional labor-2^nd^ stage of labor | 1 | 0.0  (0.0, 1.8) | 0.6  (0.0, 3.4) | 1.2  (0.2, 3.4) | 0.8  (0.1, 2.8) | 0.8  (0.2, 2.2) | -  (0.0, 1.8) | 2.0  (0.6, 5.1) | 0.7  (0.1, 2.7) | 0.0  (0.0, 3.0) | 1.5  (0.0, 8.2) | 0.8  (0.5, 1.2) |
|  | 2 | 0.0  (0.0, 1.8) | 0.0  (0.0, 2.3) | 0.8  (0.1, 2.8) | 0.8  (0.1, 2.8) | 0.5  (0.1, 1.9) | 0.0  (0.0, 1.2) | 0.5  (0.0, 2.8) | 0.7  (0.1, 2.7) | 0.8  (0.0, 4.5) | 0.0  (0.0, 5.4) | 0.5  (0.2, 0.8) |
|  | Incorrect classification | 0.0  (0.0, 1.8) | 0.0  (0.0, 2.3) | 0.4  (0.0, 2.1) | 0.0  (0.0, 1.4) | 0.8  (0.2, 2.2) | 0.0  (0.0, 1.2) | 0.0  (0.0, 1.9) | 0.0  (0.0, 1.4) | 0.0  (0.0, 3.0) | 0.0  (0.0, 5.4) | 0.2  (0.1, 0.5) |
| Gestational diabetes mellitus | 1 | 0.0  (0.0, 1.8) | 4.3  (1.8, 8.7) | 2.3  (0.9, 5.0) | 3.9  (1.9, 7.0) | 0.0  (0.0, 1.0) | -  (0.7, 4.3) | 5.1  (2.5, 9.2) | 0.7  (0.1, 2.7) | 1.7  (0.2, 5.8) | 0.0  (0.0, 5.4) | 1.9  (1.4, 2.6) |
|  | 2 | 0.0  (0.0, 1.8) | 0.0  (0.0, 2.3) | 0.0  (0.0, 1.4) | 1.2  (0.2, 3.3) | 0.0  (0.0, 1.0) | 0.0  (0.0, 1.2) | 0.0  (0.0, 1.9) | 0.0  (0.0, 1.4) | 0.0  (0.0, 3.0) | 0.0  (0.0, 5.4) | 0.1  (0.0, 0.4) |
|  | 3 | 0.0  (0.0, 1.8) | 1.9  (0.4, 5.3) | 0.0  (0.0, 1.4) | 0.4  (0.0, 2.1) | 0.8  (0.2, 2.2) | -  (0.4, 3.4) | 0.0  (0.0, 1.9) | 0.0  (0.0, 1.4) | 0.0  (0.0, 3.0) | 0.0  (0.0, 5.4) | 0.5  (0.3, 0.9) |
|  | Insufficient evidence | 0.0  (0.0, 1.8) | 0.0  (0.0, 2.3) | 0.0  (0.0, 1.4) | 0.0  (0.0, 1.4) | 0.0  (0.0, 1.0) | 0.0  (0.0, 1.2) | 0.5  (0.0, 2.8) | 0.0  (0.0, 1.4) | 0.0  (0.0, 3.0) | 0.0  (0.0, 5.4) | 0.1  (0.0, 0.3) |
| Non-reassuring fetal status | 1 | 0.0  (0.0, 1.8) | 2.5  (0.7, 6.2) | 0.4  (0.0, 2.1) | 0.0  (0.0, 1.4) | 0.8  (0.2, 2.2) | -  (0.0, 1.8) | 5.6  (2.8, 9.8) | 1.5  (0.4, 3.8) | 0.0  (0.0, 3.0) | 3.0  (0.4, 10.5) | 1.2  (0.8, 1.7) |
|  | 2 | 0.0  (0.0, 1.8) | 3.1  (1.0, 7.1) | 1.6  (0.4, 3.9) | 0.4  (0.0, 2.1) | 1.3  (0.4, 3.0) | 0.0  (0.0, 1.2) | 1.0  (0.1, 3.6) | 0.0  (0.0, 1.4) | 0.0  (0.0, 3.0) | 0.0  (0.0, 5.4) | 0.8  (0.5, 1.2) |
|  | 3 | 0.0  (0.0, 1.8) | 0.6  (0.0, 3.4) | 0.4  (0.0, 2.1) | 0.8  (0.1, 2.8) | 4.4  (2.6, 6.9) | 0.0  (0.0, 1.2) | 2.6  (0.8, 5.9) | 1.1  (0.2, 3.2) | 0.0  (0.0, 3.0) | 1.5  (0.0, 8.2) | 1.4  (0.9, 1.9) |
|  | Incorrect classification | 0.0  (0.0, 1.8) | 0.0  (0.0, 2.3) | 2.7  (1.1, 5.5) | 0.0  (0.0, 1.4) | 11.6  (8.6, 15.2) | 0.0  (0.0, 1.2) | 2.6  (0.8, 5.9) | 1.9  (0.6, 4.3) | 0.0  (0.0, 3.0) | 0.0  (0.0, 5.4) | 2.8  (2.2, 3.6) |
|  | Unavailable | 0.0  (0.0, 1.8) | 0.0  (0.0, 2.3) | 0.0  (0.0, 1.4) | 0.4  (0.0, 2.1) | 0.3  (0.0, 1.4) | 0.0  (0.0, 1.2) | 0.0  (0.0, 1.9) | 0.0  (0.0, 1.4) | 0.0  (0.0, 3.0) | 0.0  (0.0, 5.4) | 0.1  (0.0, 0.3) |
| Pathways to preterm birth | Total | 10.5  (6.6, 15.5) | 6.8  (3.4, 11.8) | 4.3  (2.2, 7.5) | 5.0  (2.7, 8.4) | 11.3  (8.4, 14.9) | -  (2.8, 8.1) | 5.6  (2.8, 9.8) | 7.1  (4.3, 10.8) | 10.7  (5.9, 17.7) | 12.1  (5.4, 22.5) | 7.5  (6.4, 8.6) |
| Preterm rupture of membranes | 1 | 0.0  (0.0, 1.8) | 1.2  (0.2, 4.4) | 0.0  (0.0, 1.4) | 0.0  (0.0, 1.4) | 0.5  (0.1, 1.9) | -  (0.1, 2.4) | 1.5  (0.3, 4.4) | 1.9  (0.6, 4.3) | 0.0  (0.0, 3.0) | 0.0  (0.0, 5.4) | 0.6  (0.3, 1.1) |
|  | 2 | 0.0  (0.0, 1.8) | 0.0  (0.0, 2.3) | 0.4  (0.0, 2.1) | 0.0  (0.0, 1.4) | 0.8  (0.2, 2.2) | 0.0  (0.0, 1.2) | 0.0  (0.0, 1.9) | 0.0  (0.0, 1.4) | 0.0  (0.0, 3.0) | 4.6  (1.0, 12.7) | 0.3  (0.1, 0.7) |
|  | 3 | 1.5  (0.3, 4.3) | 0.6  (0.0, 3.4) | 0.4  (0.0, 2.1) | 0.0  (0.0, 1.4) | 0.3  (0.0, 1.4) | -  (0.5, 3.8) | 0.5  (0.0, 2.8) | 0.0  (0.0, 1.4) | 0.8  (0.0, 4.5) | 0.0  (0.0, 5.4) | 0.6  (0.3, 1.0) |
|  | Incorrect classification | 0.0  (0.0, 1.8) | 0.0  (0.0, 2.3) | 0.4  (0.0, 2.1) | 0.0  (0.0, 1.4) | 0.8  (0.2, 2.2) | 0.0  (0.0, 1.2) | 0.0  (0.0, 1.9) | 0.4  (0.0, 2.1) | 0.0  (0.0, 3.0) | 0.0  (0.0, 5.4) | 0.2  (0.1, 0.5) |
|  | Unavailable | 0.0  (0.0, 1.8) | 0.0  (0.0, 2.3) | 0.0  (0.0, 1.4) | 0.0  (0.0, 1.4) | 0.0  (0.0, 1.0) | 0.0  (0.0, 1.2) | 0.0  (0.0, 1.9) | 0.4  (0.0, 2.1) | 0.0  (0.0, 3.0) | 0.0  (0.0, 5.4) | 0.1  (0.0, 0.3) |
| Preterm labor | 1 | 0.0  (0.0, 1.8) | 1.2  (0.2, 4.4) | 0.0  (0.0, 1.4) | 2.3  (0.9, 5.0) | 0.5  (0.1, 1.9) | -  (0.5, 3.8) | 0.5  (0.0, 2.8) | 2.6  (1.1, 5.3) | 4.1  (1.4, 9.4) | 1.5  (0.0, 8.2) | 1.3  (0.9, 1.9) |
|  | 2 | 0.0  (0.0, 1.8) | 0.6  (0.0, 3.4) | 0.8  (0.1, 2.8) | 1.2  (0.2, 3.3) | 0.8  (0.2, 2.2) | 0.0  (0.0, 1.2) | 1.0  (0.1, 3.6) | 0.4  (0.0, 2.1) | 3.3  (0.9, 8.3) | 0.0  (0.0, 5.4) | 0.7  (0.4, 1.2) |
|  | 3 | 0.0  (0.0, 1.8) | 0.6  (0.0, 3.4) | 0.8  (0.1, 2.8) | 1.2  (0.2, 3.3) | 0.3  (0.0, 1.4) | 0.0  (0.0, 1.2) | 1.0  (0.1, 3.6) | 0.4  (0.0, 2.1) | 1.7  (0.2, 5.8) | 3.0  (0.4, 10.5) | 0.6  (0.3, 1.1) |
|  | Incorrect classification | 3.0  (1.1, 6.4) | 1.2  (0.2, 4.4) | 1.6  (0.4, 3.9) | 0.0  (0.0, 1.4) | 5.2  (3.2, 7.9) | 0.0  (0.0, 1.2) | 0.5  (0.0, 2.8) | 0.7  (0.1, 2.7) | 0.8  (0.0, 4.5) | 0.0  (0.0, 5.4) | 1.6  (1.1, 2.2) |
|  | Unavailable | 1.0  (0.1, 3.6) | 0.0  (0.0, 2.3) | 0.0  (0.0, 1.4) | 0.0  (0.0, 1.4) | 0.5  (0.1, 1.9) | -  (0.1, 2.4) | 0.0  (0.0, 1.9) | 0.0  (0.0, 1.4) | 0.0  (0.0, 3.0) | 0.0  (0.0, 5.4) | 0.3  (0.1, 0.6) |
| Provider-initiated preterm birth | 1 | 5.0  (2.4, 9.0) | 1.2  (0.2, 4.4) | 0.0  (0.0, 1.4) | 0.4  (0.0, 2.1) | 1.6  (0.6, 3.3) | -  (0.0, 1.8) | 0.5  (0.0, 2.8) | 0.4  (0.0, 2.0) | 0.0  (0.0, 3.0) | 1.5  (0.0, 8.2) | 1.0  (0.7, 1.6) |
|  | 2 | 0.0  (0.0, 1.8) | 0.0  (0.0, 2.3) | 0.0  (0.0, 1.4) | 0.0  (0.0, 1.4) | 0.0  (0.0, 1.0) | 0.0  (0.0, 1.2) | 0.0  (0.0, 1.9) | 0.0  (0.0, 1.4) | 0.0  (0.0, 3.0) | 1.5  (0.0, 8.2) | 0.1  (0.0, 0.3) |
|  | 3 | 0.0  (0.0, 1.8) | 0.0  (0.0, 2.3) | 0.0  (0.0, 1.4) | 0.0  (0.0, 1.4) | 0.3  (0.0, 1.4) | 0.0  (0.0, 1.2) | 0.0  (0.0, 1.9) | 0.0  (0.0, 1.4) | 0.0  (0.0, 3.0) | 0.0  (0.0, 5.4) | 0.1  (0.0, 0.3) |

EOI, event of interest; GAIA, Global Alignment of Immunization safety Assessment in pregnancy; %, percentage of participants with the EOI; N, number of participants included in the analyses.

# Supplementary Table 7. Percentage of neonatal EOIs occurring from birth to 28 days post-delivery, according to GAIA levels of diagnostic certainty, overall and by country (neonatal analysis set)

|  |  | % (95% confidence interval) | | | | | | | | | | |
| --- | --- | --- | --- | --- | --- | --- | --- | --- | --- | --- | --- | --- |
| EOI | GAIA level | Bangladesh  (N=190) | Malaysia  (N=159) | Philippines  (N=240) | Thailand  (N=255) | South Africa  (N=373) | Argentina  (N=283) | Brazil  (N=183) | Colombia  (N=255) | Mexico  (N=95) | Panama  (N=61) | Overall  (N=2094) |
| Small for gestational age | 1 | 0.5  (0.0, 2.9) | 4.4  (1.8, 8.9) | 2.1  (0.7, 4.8) | 3.1  (1.4, 6.1) | 0.5  (0.1, 1.9) | -  (0.8, 4.6) | 4.4  (1.9, 8.4) | 2.0  (0.6, 4.5) | 4.2  (1.2, 10.4) | 0.0  (0.0, 5.9) | 2.2  (1.6, 2.9) |
|  | 2a | 5.8  (2.9, 10.1) | 1.3  (0.2, 4.5) | 2.5  (0.9, 5.4) | 0.0  (0.0, 1.4) | 1.9  (0.8, 3.8) | -  (0.0, 2.0) | 0.0  (0.0, 2.0) | 0.4  (0.0, 2.2) | 0.0  (0.0, 3.8) | 0.0  (0.0, 5.9) | 1.3  (0.9, 1.9) |
|  | 2b | 2.6  (0.9, 6.0) | 0.0  (0.0, 2.3) | 1.3  (0.3, 3.6) | 0.0  (0.0, 1.4) | 0.8  (0.2, 2.3) | 0.0  (0.0, 1.3) | 0.0  (0.0, 2.0) | 0.0  (0.0, 1.4) | 0.0  (0.0, 3.8) | 0.0  (0.0, 5.9) | 0.5  (0.3, 0.9) |
|  | 3a | 0.0  (0.0, 1.9) | 0.0  (0.0, 2.3) | 0.0  (0.0, 1.5) | 0.0  (0.0, 1.4) | 0.0  (0.0, 1.0) | 0.0  (0.0, 1.3) | 0.0  (0.0, 2.0) | 0.8  (0.1, 2.8) | 0.0  (0.0, 3.8) | 0.0  (0.0, 5.9) | 0.1  (0.0, 0.3) |
|  | 3b | 0.0  (0.0, 1.9) | 0.0  (0.0, 2.3) | 0.0  (0.0, 1.5) | 0.0  (0.0, 1.4) | 0.5  (0.1, 1.9) | 0.0  (0.0, 1.3) | 0.0  (0.0, 2.0) | 0.0  (0.0, 1.4) | 0.0  (0.0, 3.8) | 0.0  (0.0, 5.9) | 0.1  (0.0, 0.3) |
|  | Incorrect classification | 0.5  (0.0, 2.9) | 0.6  (0.0, 3.5) | 0.4  (0.0, 2.3) | 0.0  (0.0, 1.4) | 4.0  (2.3, 6.6) | 0.0  (0.0, 1.3) | 0.0  (0.0, 2.0) | 0.0  (0.0, 1.4) | 0.0  (0.0, 3.8) | 0.0  (0.0, 5.9) | 0.9  (0.5, 1.4) |
|  | Unavailable | 2.1  (0.6, 5.3) | 0.0  (0.0, 2.3) | 0.0  (0.0, 1.5) | 0.0  (0.0, 1.4) | 0.0  (0.0, 1.0) | 0.0  (0.0, 1.3) | 0.0  (0.0, 2.0) | 0.0  (0.0, 1.4) | 0.0  (0.0, 3.8) | 0.0  (0.0, 5.9) | 0.2  (0.1, 0.5) |
| Low/very low birthweight | 1 | 10.5  (6.6, 15.8) | 9.4  (5.4, 15.1) | 7.9  (4.8, 12.1) | 3.9  (1.9, 7.1) | 2.7  (1.3, 4.9) | -  (2.2, 7.3) | 3.8  (1.6, 7.7) | 2.8  (1.1, 5.6) | 4.2  (1.2, 10.4) | 3.3  (0.4, 11.4) | 5.1  (4.2, 6.1) |
|  | 2 | 0.0  (0.0, 1.9) | 1.3  (0.2, 4.5) | 2.5  (0.9, 5.4) | 0.0  (0.0, 1.4) | 0.0  (0.0, 1.0) | 0.0  (0.0, 1.3) | 0.0  (0.0, 2.0) | 0.4  (0.0, 2.2) | 1.1  (0.0, 5.7) | 1.6  (0.0, 8.8) | 0.5  (0.3, 0.9) |
|  | 3 | 0.0  (0.0, 1.9) | 1.3  (0.2, 4.5) | 2.1  (0.7, 4.8) | 0.0  (0.0, 1.4) | 2.1  (0.9, 4.2) | -  (0.0, 2.0) | 0.0  (0.0, 2.0) | 0.0  (0.0, 1.4) | 0.0  (0.0, 3.8) | 0.0  (0.0, 5.9) | 0.8  (0.4, 1.2) |
|  | 4 | 0.0  (0.0, 1.9) | 0.0  (0.0, 2.3) | 0.0  (0.0, 1.5) | 0.4  (0.0, 2.2) | 0.0  (0.0, 1.0) | 0.0  (0.0, 1.3) | 0.0  (0.0, 2.0) | 1.2  (0.2, 3.4) | 1.1  (0.0, 5.7) | 0.0  (0.0, 5.9) | 0.2  (0.1, 0.6) |
|  | Incorrect classification | 1.6  (0.3, 4.5) | 0.0  (0.0, 2.3) | 0.4  (0.0, 2.3) | 0.0  (0.0, 1.4) | 3.5  (1.9, 5.9) | -  (0.0, 2.0) | 0.0  (0.0, 2.0) | 0.0  (0.0, 1.4) | 0.0  (0.0, 3.8) | 0.0  (0.0, 5.9) | 0.9  (0.5, 1.4) |
| Neonatal encephalopathy | 1 | 0.0  (0.0, 1.9) | 0.6  (0.0, 3.5) | 0.4  (0.0, 2.3) | 0.0  (0.0, 1.4) | 0.0  (0.0, 1.0) | -  (0.0, 2.0) | 0.0  (0.0, 2.0) | 0.4  (0.0, 2.2) | 0.0  (0.0, 3.8) | 0.0  (0.0, 5.9) | 0.2  (0.1, 0.5) |
|  | 3 | 0.0  (0.0, 1.9) | 0.0  (0.0, 2.3) | 0.0  (0.0, 1.5) | 0.0  (0.0, 1.4) | 0.3  (0.0, 1.5) | 0.0  (0.0, 1.3) | 0.0  (0.0, 2.0) | 0.4  (0.0, 2.2) | 0.0  (0.0, 3.8) | 0.0  (0.0, 5.9) | 0.1  (0.0, 0.3) |
| Congenital microcephaly |  | 0.0  (0.0, 1.9) | 0.0  (0.0, 2.3) | 0.0  (0.0, 1.5) | 1.2  (0.2, 3.4) | 3.5  (1.9, 5.9) | 0.0  (0.0, 1.3) | 0.0  (0.0, 2.0) | 0.0  (0.0, 1.4) | 1.1  (0.0, 5.7) | 0.0  (0.0, 5.9) | 0.8  (0.5, 1.3) |
| Postnatally diagnosed | 1 | 0.0  (0.0, 1.9) | 0.0  (0.0, 2.3) | 0.0  (0.0, 1.5) | 0.8  (0.1, 2.8) | 1.1  (0.3, 2.7) | 0.0  (0.0, 1.3) | 0.0  (0.0, 2.0) | 0.0  (0.0, 1.4) | 1.1  (0.0, 5.7) | 0.0  (0.0, 5.9) | 0.3  (0.1, 0.7) |
|  | 2a | 0.0  (0.0, 1.9) | 0.0  (0.0, 2.3) | 0.0  (0.0, 1.5) | 0.0  (0.0, 1.4) | 0.8  (0.2, 2.3) | 0.0  (0.0, 1.3) | 0.0  (0.0, 2.0) | 0.0  (0.0, 1.4) | 0.0  (0.0, 3.8) | 0.0  (0.0, 5.9) | 0.1  (0.0, 0.4) |
|  | 2b | 0.0  (0.0, 1.9) | 0.0  (0.0, 2.3) | 0.0  (0.0, 1.5) | 0.0  (0.0, 1.4) | 1.1  (0.3, 2.7) | 0.0  (0.0, 1.3) | 0.0  (0.0, 2.0) | 0.0  (0.0, 1.4) | 0.0  (0.0, 3.8) | 0.0  (0.0, 5.9) | 0.2  (0.1, 0.5) |
|  | Incorrect classification | 0.0  (0.0, 1.9) | 0.0  (0.0, 2.3) | 0.0  (0.0, 1.5) | 0.0  (0.0, 1.4) | 0.5  (0.1, 1.9) | 0.0  (0.0, 1.3) | 0.0  (0.0, 2.0) | 0.0  (0.0, 1.4) | 0.0  (0.0, 3.8) | 0.0  (0.0, 5.9) | 0.1  (0.0, 0.3) |
| Prenatally diagnosed | Incorrect classification | 0.0  (0.0, 1.9) | 0.0  (0.0, 2.3) | 0.0  (0.0, 1.5) | 0.4  (0.0, 2.2) | 0.0  (0.0, 1.0) | 0.0  (0.0, 1.3) | 0.0  (0.0, 2.0) | 0.0  (0.0, 1.4) | 0.0  (0.0, 3.8) | 0.0  (0.0, 5.9) | 0.1  (0.0, 0.3) |
| Congenital anomalies |  | 1.6  (0.3, 4.5) | 3.8  (1.4, 8.0) | 1.7  (0.5, 4.2) | 3.9  (1.9, 7.1) | 13.7  (10.4, 17.6) | -  (2.0, 6.9) | 3.8  (1.6, 7.7) | 1.2  (0.2, 3.4) | 8.4  (3.7, 15.9) | 0.0  (0.0, 5.9) | 4.9  (4.0, 5.9) |
| Major external structural defects | 1 | 0.5  (0.0, 2.9) | 0.0  (0.0, 2.3) | 0.0  (0.0, 1.5) | 0.8  (0.1, 2.8) | 0.0  (0.0, 1.0) | -  (0.0, 2.0) | 1.1  (0.1, 3.9) | 0.4  (0.0, 2.2) | 2.1  (0.3, 7.4) | 0.0  (0.0, 5.9) | 0.4  (0.2, 0.8) |
|  | 2 | 0.0  (0.0, 1.9) | 0.0  (0.0, 2.3) | 0.0  (0.0, 1.5) | 0.0  (0.0, 1.4) | 1.3  (0.4, 3.1) | 0.0  (0.0, 1.3) | 0.0  (0.0, 2.0) | 0.0  (0.0, 1.4) | 0.0  (0.0, 3.8) | 0.0  (0.0, 5.9) | 0.2  (0.1, 0.6) |
|  | 3 | 0.0  (0.0, 1.9) | 0.0  (0.0, 2.3) | 0.0  (0.0, 1.5) | 0.0  (0.0, 1.4) | 0.8  (0.2, 2.3) | 0.0  (0.0, 1.3) | 0.0  (0.0, 2.0) | 0.0  (0.0, 1.4) | 0.0  (0.0, 3.8) | 0.0  (0.0, 5.9) | 0.1  (0.0, 0.4) |
|  | Incorrect classification | 0.0  (0.0, 1.9) | 0.0  (0.0, 2.3) | 0.0  (0.0, 1.5) | 0.0  (0.0, 1.4) | 4.8  (2.9, 7.5) | 0.0  (0.0, 1.3) | 0.0  (0.0, 2.0) | 0.0  (0.0, 1.4) | 0.0  (0.0, 3.8) | 0.0  (0.0, 5.9) | 0.9  (0.5, 1.4) |
|  | Unavailable | 0.0  (0.0, 1.9) | 0.0  (0.0, 2.3) | 0.0  (0.0, 1.5) | 0.0  (0.0, 1.4) | 0.8  (0.2, 2.3) | 0.0  (0.0, 1.3) | 0.0  (0.0, 2.0) | 0.0  (0.0, 1.4) | 0.0  (0.0, 3.8) | 0.0  (0.0, 5.9) | 0.1  (0.0, 0.4) |
| Internal structural defects | 1 | 0.0  (0.0, 1.9) | 1.3  (0.2, 4.5) | 0.0  (0.0, 1.5) | 0.0  (0.0, 1.4) | 0.5  (0.1, 1.9) | -  (0.1, 2.5) | 1.1  (0.1, 3.9) | 0.8  (0.1, 2.8) | 2.1  (0.3, 7.4) | 0.0  (0.0, 5.9) | 0.6  (0.3, 1.0) |
|  | 2 | 0.0  (0.0, 1.9) | 0.0  (0.0, 2.3) | 0.4  (0.0, 2.3) | 0.0  (0.0, 1.4) | 0.3  (0.0, 1.5) | -  (0.4, 3.6) | 0.0  (0.0, 2.0) | 0.0  (0.0, 1.4) | 3.2  (0.7, 9.0) | 0.0  (0.0, 5.9) | 0.4  (0.2, 0.8) |
|  | 3 | 0.5  (0.0, 2.9) | 0.0  (0.0, 2.3) | 0.4  (0.0, 2.3) | 0.0  (0.0, 1.4) | 4.0.0  (2.3, 6.6) | -  (0.1, 2.5) | 1.1  (0.1, 3.9) | 0.0  (0.0, 1.4) | 1.1  (0.0, 5.7) | 0.0  (0.0, 5.9) | 1.1  (0.7, 1.6) |
|  | Incorrect classification | 0.0  (0.0, 1.9) | 0.0  (0.0, 2.3) | 0.0  (0.0, 1.5) | 0.0  (0.0, 1.4) | 0.5  (0.1, 1.9) | 0.0  (0.0, 1.3) | 0.0  (0.0, 2.0) | 0.0  (0.0, 1.4) | 0.0  (0.0, 3.8) | 0.0  (0.0, 5.9) | 0.1  (0.0, 0.3) |
|  | Unavailable | 0.0  (0.0, 1.9) | 0.0  (0.0, 2.3) | 0.4  (0.0, 2.3) | 0.0  (0.0, 1.4) | 0.0  (0.0, 1.0) | 0.0  (0.0, 1.3) | 0.0  (0.0, 2.0) | 0.0  (0.0, 1.4) | 0.0  (0.0, 3.8) | 0.0  (0.0, 5.9) | 0.1  (0.0, 0.3) |
| Functional defects | 1 | 0.5  (0.0, 2.9) | 1.9  (0.4, 5.4) | 0.4  (0.0, 2.3) | 2.4  (0.9, 5.1) | 0.3  (0.0, 1.5) | -  (0.0, 2.0) | 0.6  (0.0, 3.0) | 0.0  (0.0, 1.4) | 0.0  (0.0, 3.8) | 0.0  (0.0, 5.9) | 0.7  (0.4, 1.1) |
|  | 2 | 0.0  (0.0, 1.9) | 0.6  (0.0, 3.5) | 0.0  (0.0, 1.5) | 0.4  (0.0, 2.2) | 0.0  (0.0, 1.0) | -  (0.0, 2.0) | 0.0  (0.0, 2.0) | 0.0  (0.0, 1.4) | 0.0  (0.0, 3.8) | 0.0  (0.0, 5.9) | 0.1  (0.0, 0.4) |
|  | 4 | 0.0  (0.0, 1.9) | 0.0  (0.0, 2.3) | 0.0  (0.0, 1.5) | 0.4  (0.0, 2.2) | 0.0  (0.0, 1.0) | 0.0  (0.0, 1.3) | 0.0  (0.0, 2.0) | 0.0  (0.0, 1.4) | 0.0  (0.0, 3.8) | 0.0  (0.0, 5.9) | 0.1  (0.0, 0.3) |
|  | Incorrect classification | 0.0  (0.0, 1.9) | 0.0  (0.0, 2.3) | 0.0  (0.0, 1.5) | 0.0  (0.0, 1.4) | 0.3  (0.0, 1.5) | 0.0  (0.0, 1.3) | 0.0  (0.0, 2.0) | 0.0  (0.0, 1.4) | 0.0  (0.0, 3.8) | 0.0  (0.0, 5.9) | 0.1  (0.0, 0.3) |
| Neonatal death |  | 1.6  (0.3, 4.5) | 0.0  (0.0, 2.3) | 0.8  (0.1, 3.0) | 0.0  (0.0, 1.4) | 1.1  (0.3, 2.7) | 0.0  (0.0, 1.3) | 0.0  (0.0, 2.0) | 0.0  (0.0, 1.4) | 1.1  (0.0, 5.7) | 0.0  (0.0, 5.9) | 0.5  (0.2, 0.9) |
| Neonatal death in a preterm livebirth | 1 | 0.0  (0.0, 1.9) | 0.0  (0.0, 2.3) | 0.4  (0.0, 2.3) | 0.0  (0.0, 1.4) | 0.3  (0.0, 1.5) | 0.0  (0.0, 1.3) | 0.0  (0.0, 2.0) | 0.0  (0.0, 1.4) | 0.0  (0.0, 3.8) | 0.0  (0.0, 5.9) | 0.1  (0.0, 0.3) |
|  | 2 | 0.5  (0.0, 2.9) | 0.0  (0.0, 2.3) | 0.0  (0.0, 1.5) | 0.0  (0.0, 1.4) | 0.0  (0.0, 1.0) | 0.0  (0.0, 1.3) | 0.0  (0.0, 2.0) | 0.0  (0.0, 1.4) | 0.0  (0.0, 3.8) | 0.0  (0.0, 5.9) | 0.1  (0.0, 0.3) |
| Neonatal death in a term livebirth | 1 | 0.0  (0.0, 1.9) | 0.0  (0.0, 2.3) | 0.4  (0.0, 2.3) | 0.0  (0.0, 1.4) | 0.3  (0.0, 1.5) | 0.0  (0.0, 1.3) | 0.0  (0.0, 2.0) | 0.0  (0.0, 1.4) | 1.1  (0.0, 5.7) | 0.0  (0.0, 5.9) | 0.1  (0.0, 0.4) |
|  | 2 | 1.1  (0.1, 3.8) | 0.0  (0.0, 2.3) | 0.0  (0.0, 1.5) | 0.0  (0.0, 1.4) | 0.3  (0.0, 1.5) | 0.0  (0.0, 1.3) | 0.0  (0.0, 2.0) | 0.0  (0.0, 1.4) | 0.0  (0.0, 3.8) | 0.0  (0.0, 5.9) | 0.1  (0.0, 0.4) |
|  | Incorrect classification | 0.0  (0.0, 1.9) | 0.0  (0.0, 2.3) | 0.0  (0.0, 1.5) | 0.0  (0.0, 1.4) | 0.3  (0.0, 1.5) | 0.0  (0.0, 1.3) | 0.0  (0.0, 2.0) | 0.0  (0.0, 1.4) | 0.0  (0.0, 3.8) | 0.0  (0.0, 5.9) | 0.1  (0.0, 0.3) |
| Neonatal infections |  | 6.3  (3.3, 10.8) | 1.3  (0.2, 4.5) | 3.3  (1.5, 6.5) | 1.6  (0.4, 4.0) | 5.4  (3.3, 8.2) | 0.0  (0.0, 1.3) | 6.0.0  (3.0, 10.5) | 2.4  (0.9, 5.1) | 1.1  (0.0, 5.7) | 8.2  (2.7, 18.1) | 3.3  (2.6, 4.2) |
| Blood stream infections | 1 | 0.0  (0.0, 1.9) | 0.6  (0.0, 3.5) | 0.0  (0.0, 1.5) | 0.0  (0.0, 1.4) | 0.5  (0.1, 1.9) | 0.0  (0.0, 1.3) | 0.6  (0.0, 3.0) | 0.4  (0.0, 2.2) | 1.1  (0.0, 5.7) | 1.6  (0.0, 8.8) | 0.3  (0.1, 0.7) |
|  | 2 | 1.1  (0.1, 3.8) | 0.0  (0.0, 2.3) | 0.4  (0.0, 2.3) | 0.0  (0.0, 1.4) | 0.3  (0.0, 1.5) | 0.0  (0.0, 1.3) | 1.6  (0.3, 4.7) | 0.4  (0.0, 2.2) | 0.0  (0.0, 3.8) | 1.6  (0.0, 8.8) | 0.4  (0.2, 0.8) |
|  | 3 | 0.0  (0.0, 1.9) | 0.0  (0.0, 2.3) | 0.8  (0.1, 3.0) | 0.4  (0.0, 2.2) | 0.3  (0.0, 1.5) | 0.0  (0.0, 1.3) | 0.6  (0.0, 3.0) | 0.0  (0.0, 1.4) | 0.0  (0.0, 3.8) | 1.6  (0.0, 8.8) | 0.3  (0.1, 0.6) |
|  | Incorrect classification | 0.0  (0.0, 1.9) | 0.0  (0.0, 2.3) | 0.4  (0.0, 2.3) | 0.0  (0.0, 1.4) | 0.8  (0.2, 2.3) | 0.0  (0.0, 1.3) | 1.6  (0.3, 4.7) | 0.0  (0.0, 1.4) | 0.0  (0.0, 3.8) | 1.6  (0.0, 8.8) | 0.4  (0.2, 0.8) |
|  | Unavailable | 0.5  (0.0, 2.9) | 0.0  (0.0, 2.3) | 0.0  (0.0, 1.5) | 0.0  (0.0, 1.4) | 0.5  (0.1, 1.9) | 0.0  (0.0, 1.3) | 0.0  (0.0, 2.0) | 0.0  (0.0, 1.4) | 0.0  (0.0, 3.8) | 0.0  (0.0, 5.9) | 0.1  (0.0, 0.4) |
| Respiratory infection | 1 | 0.0  (0.0, 1.9) | 0.0  (0.0, 2.3) | 0.0  (0.0, 1.5) | 0.8  (0.1, 2.8) | 0.0  (0.0, 1.0) | 0.0  (0.0, 1.3) | 0.0  (0.0, 2.0) | 0.4  (0.0, 2.2) | 0.0  (0.0, 3.8) | 0.0  (0.0, 5.9) | 0.1  (0.0, 0.4) |
|  | 2 | 1.1  (0.1, 3.8) | 0.0  (0.0, 2.3) | 0.8  (0.1, 3.0) | 0.4  (0.0, 2.2) | 0.3  (0.0, 1.5) | 0.0  (0.0, 1.3) | 0.6  (0.0, 3.0) | 0.8  (0.1, 2.8) | 0.0  (0.0, 3.8) | 0.0  (0.0, 5.9) | 0.4  (0.2, 0.8) |
|  | 3 | 3.2  (1.2, 6.8) | 0.6  (0.0, 3.5) | 0.8  (0.1, 3.0) | 0.0  (0.0, 1.4) | 0.3  (0.0, 1.5) | 0.0  (0.0, 1.3) | 1.1  (0.1, 3.9) | 0.4  (0.0, 2.2) | 0.0  (0.0, 3.8) | 0.0  (0.0, 5.9) | 0.6  (0.3, 1.1) |
|  | Incorrect classification | 0.5  (0.0, 2.9) | 0.0  (0.0, 2.3) | 0.0  (0.0, 1.5) | 0.0  (0.0, 1.4) | 2.4  (1.1, 4.5) | 0.0  (0.0, 1.3) | 0.0  (0.0, 2.0) | 0.0  (0.0, 1.4) | 0.0  (0.0, 3.8) | 0.0  (0.0, 5.9) | 0.5  (0.2, 0.9) |
|  | Unavailable | 0.0  (0.0, 1.9) | 0.0  (0.0, 2.3) | 0.0  (0.0, 1.5) | 0.0  (0.0, 1.4) | 0.0  (0.0, 1.0) | 0.0  (0.0, 1.3) | 0.0  (0.0, 2.0) | 0.0  (0.0, 1.4) | 0.0  (0.0, 3.8) | 1.6  (0.0, 8.8) | 0.1  (0.0, 0.3) |
| Respiratory distress | 1 | 1.6  (0.3, 4.5) | 3.8  (1.4, 8.0) | 2.5  (0.9, 5.4) | 2.4  (0.9, 5.1) | 1.3  (0.4, 3.1) | -  (0.2, 3.1) | 8.7  (5.1, 13.8) | 2.4  (0.9, 5.1) | 6.3  (2.4, 13.2) | 3.3  (0.4, 11.4) | 2.8  (2.2, 3.6) |
|  | 2 | 0.0  (0.0, 1.9) | 0.6  (0.0, 3.5) | 0.0  (0.0, 1.5) | 0.0  (0.0, 1.4) | 1.6  (0.6, 3.5) | -  (0.0, 2.0) | 1.1  (0.1, 3.9) | 1.6  (0.4, 4.0) | 0.0  (0.0, 3.8) | 0.0  (0.0, 5.9) | 0.7  (0.4, 1.1) |
|  | 3 | 0.0  (0.0, 1.9) | 0.0  (0.0, 2.3) | 0.0  (0.0, 1.5) | 0.0  (0.0, 1.4) | 0.5  (0.1, 1.9) | 0.0  (0.0, 1.3) | 0.0  (0.0, 2.0) | 0.0  (0.0, 1.4) | 0.0  (0.0, 3.8) | 0.0  (0.0, 5.9) | 0.1  (0.0, 0.3) |
|  | 4 | 0.0  (0.0, 1.9) | 0.0  (0.0, 2.3) | 0.0  (0.0, 1.5) | 0.0  (0.0, 1.4) | 0.3  (0.0, 1.5) | -  (0.0, 2.0) | 2.2  (0.6, 5.5) | 1.6  (0.4, 4.0) | 1.1  (0.0, 5.7) | 0.0  (0.0, 5.9) | 0.5  (0.3, 0.9) |
|  | Incorrect classification | 0.0  (0.0, 1.9) | 0.0  (0.0, 2.3) | 0.0  (0.0, 1.5) | 0.0  (0.0, 1.4) | 2.1  (0.9, 4.2) | 0.0  (0.0, 1.3) | 0.0  (0.0, 2.0) | 0.0  (0.0, 1.4) | 0.0  (0.0, 3.8) | 0.0  (0.0, 5.9) | 0.4  (0.2, 0.8) |
| Preterm birth | 1 | 1.6  (0.3, 4.5) | 8.2  (4.4, 13.6) | 2.9  (1.2, 5.9) | 3.1  (1.4, 6.1) | 0.3  (0.0, 1.5) | -  (2.7, 8.2) | 3.8  (1.6, 7.7) | 5.1  (2.7, 8.6) | 3.2  (0.7, 9.0) | 6.6  (1.8, 16.0) | 3.5  (2.7, 4.4) |
|  | 2a | 7.4  (4.1, 12.1) | 0.0  (0.0, 2.3) | 0.8  (0.1, 3.0) | 1.2  (0.2, 3.4) | 4.8  (2.9, 7.5) | -  (0.1, 2.5) | 1.1  (0.1, 3.9) | 0.0  (0.0, 1.4) | 1.1  (0.0, 5.7) | 1.6  (0.0, 8.8) | 2.1  (1.5, 2.8) |
|  | 2b | 3.7  (1.5, 7.4) | 0.0  (0.0, 2.3) | 0.0  (0.0, 1.5) | 0.8  (0.1, 2.8) | 2.4  (1.1, 4.5) | 0.0  (0.0, 1.3) | 0.0  (0.0, 2.0) | 0.0  (0.0, 1.4) | 1.1  (0.0, 5.7) | 0.0  (0.0, 5.9) | 0.9  (0.6, 1.4) |
|  | 3a | 0.0  (0.0, 1.9) | 0.0  (0.0, 2.3) | 0.4  (0.0, 2.3) | 0.4  (0.0, 2.2) | 0.0  (0.0, 1.0) | 0.0  (0.0, 1.3) | 0.0  (0.0, 2.0) | 0.0  (0.0, 1.4) | 0.0  (0.0, 3.8) | 0.0  (0.0, 5.9) | 0.1  (0.0, 0.3) |
|  | 3b | 0.0  (0.0, 1.9) | 0.0  (0.0, 2.3) | 0.4  (0.0, 2.3) | 0.0  (0.0, 1.4) | 0.0  (0.0, 1.0) | 0.0  (0.0, 1.3) | 0.0  (0.0, 2.0) | 0.0  (0.0, 1.4) | 0.0  (0.0, 3.8) | 0.0  (0.0, 5.9) | 0.1  (0.0, 0.3) |
|  | Incorrect classification | 0.0  (0.0, 1.9) | 0.0  (0.0, 2.3) | 0.0  (0.0, 1.5) | 0.0  (0.0, 1.4) | 0.8  (0.2, 2.3) | 0.0  (0.0, 1.3) | 0.0  (0.0, 2.0) | 0.0  (0.0, 1.4) | 0.0  (0.0, 3.8) | 0.0  (0.0, 5.9) | 0.1  (0.0, 0.4) |
| Failure to thrive | 1 | 0.5  (0.0, 2.9) | 0.0  (0.0, 2.3) | 0.8  (0.1, 3.0) | 0.4  (0.0, 2.2) | 0.0  (0.0, 1.0) | 0.0  (0.0, 1.3) | 0.0  (0.0, 2.0) | 0.0  (0.0, 1.4) | 0.0  (0.0, 3.8) | 0.0  (0.0, 5.9) | 0.2  (0.1, 0.5) |
|  | Incorrect classification | 0.0  (0.0, 1.9) | 0.6  (0.0, 3.5) | 0.0  (0.0, 1.5) | 0.0  (0.0, 1.4) | 0.5  (0.1, 1.9) | 0.0  (0.0, 1.3) | 0.0  (0.0, 2.0) | 0.0  (0.0, 1.4) | 0.0  (0.0, 3.8) | 0.0  (0.0, 5.9) | 0.1  (0.0, 0.4) |

EOI, event of interest; GAIA, Global Alignment of Immunization safety Assessment in pregnancy; %, percentage of participants with the EOI; N, number of participants included in the analyses.

# Supplementary Table 8. Risk factors^a^ for pregnancy-related events of interest identified through univariate logistic regression (maternal analysis set)

| Event of interest/Risk factor | Odds ratio (95% CI) | p-value |
| --- | --- | --- |
| Hypertensive disorders of pregnancy |  |  |
| Prenatal smoking exposure during this pregnancy | 1.75 (0.94, 3.27) | 0.0773 |
| BMI pre-pregnancy (kg/cm^2^) | 1.16 (1.12, 1.21) | <0.0001 |
| Mother currently lives in a country or region with Zika transmission | 0.47 (0.32, 0.7) | 0.0002 |
| Cesarean section in previous pregnancy | 2.14 (1.25, 3.68) | 0.0055 |
| Gestational hypertension |  |  |
| BMI pre-pregnancy (kg/cm^2^) | 1.21 (1.15, 1.26) | <0.0001 |
| Mother currently lives in a country or region with Zika transmission | 0.47 (0.29, 0.78) | 0.0033 |
| Cesarean section in previous pregnancy | 2.86 (1.43, 5.73) | 0.0030 |
| Pre-eclampsia |  |  |
| Prenatal smoking exposure during this pregnancy | 2.53 (0.97, 6.61) | 0.0578 |
| BMI pre-pregnancy (kg/cm^2^) | 1.12 (1.05, 1.19) | 0.0008 |
| Mother currently lives in a country or region with Zika transmission | 0.44 (0.22, 0.87) | 0.0187 |
| Pre-eclampsia with severe features (including eclampsia) |  |  |
| Cesarean section in previous pregnancy | 3.96 (0.88, 17.78) | 0.0728 |
| Antenatal bleeding |  |  |
| Prenatal smoking exposure during this pregnancy | 6.05 (1.59, 23.07) | 0.0084 |
| Alcohol consumption during this pregnancy | 4.87 (1.28, 18.54) | 0.0203 |
| Mother currently lives in a country or region with Zika transmission | 0.29 (0.09, 0.95) | 0.0411 |
| Placental abruption |  |  |
| Prenatal smoking exposure during this pregnancy | 6.92 (1.77, 27.06) | 0.0055 |
| Alcohol consumption during this pregnancy | 5.57 (1.43, 21.74) | 0.0135 |
| Mother currently lives in a country or region with Zika transmission | 0.24 (0.07, 0.83) | 0.0248 |
| Postpartum hemorrhage |  |  |
| Mother currently lives in a country or region with Zika transmission | 0.44 (0.22, 0.87) | 0.0187 |
| Dysfunctional labor |  |  |
| BMI pre-pregnancy (kg/cm^2^) | 1.06 (1.01, 1.11) | 0.0127 |
| First stage of labor |  |  |
| BMI pre-pregnancy (kg/cm^2^) | 1.05 (0.99, 1.12) | 0.0778 |
| Second stage of labor |  |  |
| BMI pre-pregnancy (kg/cm^2^) | 1.07 (0.99, 1.15) | 0.0799 |
| Gestational diabetes mellitus |  |  |
| BMI pre-pregnancy (kg/cm^2^) | 1.08 (1.03, 1.14) | 0.0029 |
| Mother currently lives in a country or region with Zika transmission | 3.35 (1.21, 9.29) | 0.0204 |
| Cesarean section in previous pregnancy | 3.04 (1.5, 6.16) | 0.0020 |
| Highest education level of mother^b^ | 2.21 (1.29, 3.8) | 0.0039 |
| Non-reassuring fetal status |  |  |
| Prenatal smoking exposure during this pregnancy | 1.73 (0.95, 3.16) | 0.0733 |
| BMI pre-pregnancy (kg/cm^2^) | 1.1 (1.06, 1.14) | <0.0001 |
| Mother currently lives in a country or region with Zika transmission | 0.19 (0.13, 0.27) | <0.0001 |
| Highest education level of mother^b^ | 0.59 (0.36, 0.95) | 0.0316 |
| Pathways to preterm birth |  |  |
| Prenatal smoking exposure during this pregnancy | 2.05 (1.21, 3.46) | 0.0072 |
| Mother currently lives in a country or region with Zika transmission | 0.64 (0.44, 0.91) | 0.0135 |
| Cesarean section in previous pregnancy | 1.83 (1.16, 2.88) | 0.0099 |
| Preterm labor |  |  |
| Prenatal smoking exposure during this pregnancy | 2.51 (1.36, 4.63) | 0.0031 |
| Mother currently lives in a country or region with Zika transmission | 0.59 (0.38, 0.91) | 0.0184 |
| Provider-initiated preterm birth |  |  |
| Cesarean section in previous pregnancy | 13.24 (3.75, 46.79) | <0.0001 |
| Oligohydramnios |  |  |
| Mother currently lives in a country or region with Zika transmission | 4.94 (1.19, 20.53) | 0.0279 |
| Any other pregnancy-related event considered by the investigator to be of concern | |  |
| Prenatal smoking exposure during this pregnancy | 3.18 (1.96, 5.16) | <0.0001 |
| Mother currently lives in a country or region with Zika transmission | 0.4 (0.28, 0.57) | <0.0001 |
| Highest education level of mother^b^ | 0.54 (0.34, 0.87) | 0.0111 |

CI, confidence interval; BMI, body mass index.

Notes:

^a^Only risk factors reaching statistical significance with p-value ≤0.1 are reported.

^b^Bachelor’s degree or higher.

# Supplementary Table 9. Risk factors^a^ for pregnancy-related events of interest identified through multivariate logistic regression (maternal analysis set)

| Event of interest/Risk factor | Odds ratio (95% CI) | p-value |
| --- | --- | --- |
| Hypertensive disorders of pregnancy |  |  |
| BMI pre-pregnancy (kg/cm^2^) | 1.16 (1.1, 1.22) | <0.0001 |
| Cesarean section in previous pregnancy | 2.25 (1.27, 4) | 0.0054 |
| Gestational hypertension |  |  |
| BMI pre-pregnancy (kg/cm^2^) | 1.17 (1.09, 1.26) | <0.0001 |
| Cesarean section in previous pregnancy | 3.14 (1.49, 6.61) | 0.0026 |
| Pre-eclampsia |  |  |
| BMI pre-pregnancy (kg/cm^2^) | 1.1 (1.03, 1.18) | 0.0030 |
| Postpartum hemorrhage |  |  |
| Mother currently lives in a country or region with Zika transmission | 0.44 (0.22, 0.87) | 0.0187 |
| Dysfunctional labor |  |  |
| BMI pre-pregnancy (kg/cm^2^) | 1.06 (1.01, 1.11) | 0.0127 |
| Gestational diabetes mellitus |  |  |
| Cesarean section in previous pregnancy | 2.35 (1.13, 4.89) | 0.0224 |
| Highest education level of mother^b^ | 2.29 (1.05, 5.01) | 0.0380 |
| Non-reassuring fetal status |  |  |
| BMI pre-pregnancy (kg/cm^2^) | 1.07 (1.03, 1.11) | 0.0002 |
| Mother currently lives in a country or region with Zika transmission | 0.21 (0.15, 0.31) | <0.0001 |
| Pathways to preterm birth |  |  |
| Cesarean section in previous pregnancy | 1.98 (1.24, 3.17) | 0.0044 |
| Preterm labor |  |  |
| Prenatal smoking exposure during this pregnancy | 2.15 (1.14, 4.07) | 0.0185 |
| Provider-initiated preterm birth |  |  |
| Cesarean section in previous pregnancy | 13.24 (3.75, 46.79) | <0.0001 |
| Oligohydramnios |  |  |
| Mother currently lives in a country or region with Zika transmission | 4.94 (1.19, 20.53) | 0.0279 |
| Any other pregnancy-related event considered by the investigator to be of concern | |  |
| Prenatal smoking exposure during this pregnancy | 2.33 (1.4, 3.86) | 0.0011 |
| Mother currently lives in a country or region with Zika transmission | 0.49 (0.34, 0.7) | 0.0001 |

CI, confidence interval; BMI, body mass index.

Notes:

^a^Only risk factors reaching statistical significance with p-value ≤0.05 are reported.

^b^Bachelor’s degree or higher.

# Supplementary Table 10. Risk factors^a^ for neonatal events of interest identified through univariate logistic regression (neonatal analysis set)

| Event of interest/Risk factor | Odds ratio (95% CI) | p-value |
| --- | --- | --- |
| Small for gestational age |  |  |
| Prenatal smoking exposure during this pregnancy | 4.05 (2.42, 6.76) | <0.0001 |
| BMI pre-pregnancy (kg/cm^2^) | 0.94 (0.89, 0.98) | 0.0079 |
| Fetal growth restriction | 21.78 (8.23, 57.63) | <0.0001 |
| Low birthweight including very low birthweight |  |  |
| Prenatal smoking exposure during this pregnancy | 2.01 (1.17, 3.44) | 0.0112 |
| BMI pre-pregnancy (kg/cm^2^) | 0.96 (0.93, 1.0) | 0.0628 |
| Fetal growth restriction | 14.77 (5.62, 38.85) | <0.0001 |
| Dysfunctional labor | 0.15 (0.02, 1.1) | 0.0619 |
| Congenital microcephaly |  |  |
| Prenatal smoking exposure during this pregnancy | 8.67 (3.15, 23.82) | <0.0001 |
| Alcohol consumption during this pregnancy | 4.04 (1.3, 12.55) | 0.0157 |
| Fetal growth restriction | 8.05 (1.01, 64.43) | 0.0492 |
| Congenital microcephaly postnatally diagnosed |  |  |
| Prenatal smoking exposure during this pregnancy | 9.53 (3.41, 26.66) | <0.0001 |
| Alcohol consumption during this pregnancy | 4.38 (1.39, 13.74) | 0.0114 |
| Fetal growth restriction | 8.59 (1.07, 68.99) | 0.0430 |
| Congenital anomalies |  |  |
| Prenatal smoking exposure during this pregnancy | 4.43 (2.64, 7.42) | <0.0001 |
| BMI pre-pregnancy (kg/cm^2^) | 1.05 (1.0, 1.09) | 0.0295 |
| Gestational hypertension in current pregnancy | 8.02 (2.47, 26.01) | 0.0005 |
| Major external structural defects |  |  |
| Prenatal smoking exposure during this pregnancy | 7.65 (3.76, 15.53) | <0.0001 |
| BMI pre-pregnancy (kg/cm^2^) | 1.09 (1.02, 1.16) | 0.0099 |
| Gestational hypertension in current pregnancy | 11.37 (2.4, 53.74) | 0.0022 |
| Internal structural defects |  |  |
| Prenatal smoking exposure during this pregnancy | 3.91 (1.84, 8.28) | 0.0004 |
| Fetal growth restriction | 6.17 (1.37, 27.77) | 0.0178 |
| Functional defects |  |  |
| Gestational hypertension in current pregnancy | 10.43 (1.28, 85.05) | 0.0286 |
| Neonatal death |  |  |
| Female neonate | 0.27 (0.06, 1.27) | 0.0977 |
| Blood stream infections |  |  |
| Age of mother at delivery ≥35 years | 2.03 (0.9, 4.58) | 0.0878 |
| Gestational hypertension in current pregnancy | 5.83 (0.73, 46.47) | 0.0962 |
| Respiratory distress in the neonate |  |  |
| Alcohol consumption during this pregnancy | 1.96 (1.04, 3.68) | 0.0365 |
| BMI pre-pregnancy (kg/cm^2^) | 1.05 (1.0, 1.1) | 0.0324 |
| Female neonate | 0.63 (0.41, 0.97) | 0.0340 |
| Preterm birth |  |  |
| Dysfunctional labor | 0.17 (0.02, 1.23) | 0.0788 |
| Large for gestational age |  |  |
| BMI pre-pregnancy (kg/cm^2^) | 1.12 (1.06, 1.18) | <0.0001 |
| Macrosomia |  |  |
| BMI pre-pregnancy (kg/cm^2^) | 1.13 (1.07, 1.19) | <0.0001 |
| Any other neonatal event considered by the investigator to be of concern | |  |
| Prenatal smoking exposure during this pregnancy | 2.53 (1.61, 3.97) | <0.0001 |
| Gestational hypertension in current pregnancy | 6.44 (2.02, 20.46) | 0.0016 |
| Dysfunctional labor | 1.76 (0.95, 3.24) | 0.0701 |

CI, confidence interval; BMI, body mass index.

Note: ^a^Only risk factors reaching statistical significance with p-value ≤0.1 are reported.

# Supplementary Table 11. Risk factors^a^ for neonatal events of interest identified through multivariate logistic regression (neonatal analysis set)

| Event of interest/Risk factor | Odds ratio (95% CI) | p-value |
| --- | --- | --- |
| Small for gestational age |  |  |
| Prenatal smoking exposure during this pregnancy | 4.57 (2.7, 7.75) | <0.0001 |
| BMI pre-pregnancy (kg/cm^2^) | 0.92 (0.88, 0.97) | 0.0016 |
| Fetal growth restriction | 27.58 (10.08, 75.47) | <0.0001 |
| Low birthweight including very low birthweight |  |  |
| Prenatal smoking exposure during this pregnancy | 2.1 (1.21, 3.63) | 0.0080 |
| BMI pre-pregnancy (kg/cm^2^) | 0.96 (0.92, 1.0) | 0.0454 |
| Fetal growth restriction | 17.12 (6.32, 46.39) | <0.0001 |
| Congenital microcephaly |  |  |
| Prenatal smoking exposure during this pregnancy | 7.14 (2.35, 21.72) | 0.0005 |
| Fetal growth restriction | 8.88 (1.06, 74.44) | 0.0441 |
| Congenital microcephaly postnatally diagnosed |  |  |
| Prenatal smoking exposure during this pregnancy | 7.76 (2.5, 24.08) | 0.0004 |
| Fetal growth restriction | 9.64 (1.14, 81.65) | 0.0377 |
| Congenital anomalies |  |  |
| Prenatal smoking exposure during this pregnancy | 4.3 (2.55, 7.25) | <0.0001 |
| Gestational hypertension in current pregnancy | 6.32 (1.86, 21.47) | 0.0031 |
| Major external structural defects |  |  |
| Prenatal smoking exposure during this pregnancy | 7.07 (3.45, 14.48) | <0.0001 |
| BMI pre-pregnancy (kg/cm^2^) | 1.07 (1, 1.14) | 0.0362 |
| Gestational hypertension in current pregnancy | 7.6 (1.5, 38.47) | 0.0142 |
| Internal structural defects |  |  |
| Prenatal smoking exposure during this pregnancy | 3.94 (1.85, 8.38) | 0.0004 |
| Fetal growth restriction | 6.37 (1.39, 29.23) | 0.0172 |
| Functional defects |  |  |
| Gestational hypertension in current pregnancy | 10.43 (1.28, 85.05) | 0.0286 |
| Respiratory distress in the neonate |  |  |
| Female neonate | 0.65 (0.42, 1.0) | 0.0499 |
| Large for gestational age |  |  |
| BMI pre-pregnancy (kg/cm^2^) | 1.12 (1.06, 1.18) | <0.0001 |
| Macrosomia |  |  |
| BMI pre-pregnancy (kg/cm^2^) | 1.13 (1.07, 1.19) | <0.0001 |
| Any other neonatal event considered by the investigator to be of concern |  |  |
| Prenatal smoking exposure during this pregnancy | 2.51 (1.59, 3.96) | <0.0001 |
| Gestational hypertension in current pregnancy | 6.12 (1.89, 19.81) | 0.0025 |
| Dysfunctional labor | 1.86 (1.01, 3.44) | 0.0478 |

CI, confidence interval; BMI, body mass index.

Note: ^a^Only risk factors reaching statistical significance with p-value ≤0.05 are reported.
